# Monitoring and Predicting Health Status in Neurological Patients: The ALAMEDA Data Collection Protocol

**DOI:** 10.3390/healthcare11192656

**Published:** 2023-09-29

**Authors:** Alexandru Sorici, Lidia Băjenaru, Irina Georgiana Mocanu, Adina Magda Florea, Panagiotis Tsakanikas, Athena Cristina Ribigan, Ludovico Pedullà, Anastasia Bougea

**Affiliations:** 1 AI-MAS Laboratory, National University of Science and Technology Politehnica Bucharest, 060042 Bucharest, Romania; lidia.bajenaru@upb.ro (L.B.); irina.mocanu@upb.ro (I.G.M.); adina.florea@upb.ro (A.M.F.); 2 Institute of Communication and Computer Systems, National Technical University of Athens, 10682 Athens, Greece; ptsakanikas@cn.ntua.gr; 3 Department of Neurology, University Emergency Hospital Bucharest, 050098 Bucharest, Romania; athena_mergeani@yahoo.com; 4 Department of Neurology, Faculty of Medicine, University of Medicine and Pharmacy “Carol Davila”, 050474 Bucharest, Romania; 5 Scientific Research Area, Italian Multiple Sclerosis Foundation, 16149 Genoa, Italy; ludovico.pedulla@aism.it; 6 1st Department of Neurology, Eginition Hospital, National and Kapodistrian University of Athens, 11528 Athens, Greece; annita139@yahoo.gr

**Keywords:** PD, MS, stroke, patient reported outcomes, wearables, quantitative motor analysis, sleep analysis, mood estimation

## Abstract

(1) Objective: We explore the predictive power of a novel stream of patient data, combining wearable devices and patient reported outcomes (PROs), using an AI-first approach to classify the health status of Parkinson’s disease (PD), multiple sclerosis (MS) and stroke patients (collectively named PMSS). (2) Background: Recent studies acknowledge the burden of neurological disorders on patients and on the healthcare systems managing them. To address this, effort is invested in the digital transformation of health provisioning for PMSS patients. (3) Methods: We introduce the data collection journey within the ALAMEDA project, which continuously collects PRO data for a year through mobile applications and supplements them with data from minimally intrusive wearable devices (accelerometer bracelet, IMU sensor belt, ground force measuring insoles, and sleep mattress) worn for 1–2 weeks at each milestone. We present the data collection schedule and its feasibility, the mapping of medical predictor variables to wearable device capabilities and mobile application functionality. (4) Results: A novel combination of wearable devices and smartphone applications required for the desired analysis of motor, sleep, emotional and quality-of-life outcomes is introduced. AI-first analysis methods are presented that aim to uncover the prediction capability of diverse longitudinal and cross-sectional setups (in terms of standard medical test targets). Mobile application development and usage schedule facilitates the retention of patient engagement and compliance with the study protocol.

## 1. Introduction

Recent studies acknowledge the burden that neurological disorders have on the lives of people experiencing them, as well as on the societies and economic systems in which they live [1]. The overall direct costs of brain disorders make up for 60% of the total costs—which the European Brain Council (EBC) estimated at EUR 800 billion per year in Europe [2]. At the same time, according to the World Health Organization, there is a shortage of 4.3 million physicians, nurses and other healthcare professionals worldwide, and the gap is widening. The need arises to put in comprehensive efforts to establish policies, financing resources and improvements in healthcare services for patients of neurological diseases [3,4]. This includes empowering the healthcare providers in providing their services in the most informed manner, by being easily, and in a timely manner, aware of changes in the health status of patients.

In the case of brain disease research, technological advances and efforts towards the digital transformation of health provisioning services have shown particular promise [5,6,7]. Data analytics tools and machine learning (ML) methods can provide clinically actionable information that can complement or even empower medical recommendations [8,9].

In this context, we introduce the ALAMEDA project (https://alamedaproject.eu, accessed on 11 August 2023), whose vision is to research and prototype artificial intelligence (AI)-enabled healthcare support systems for people with brain diseases and disorders, specifically focusing on the needs of Parkinson’s disease (PD), multiple sclerosis (MS) and stroke (set of diseases hereafter collectively named PMSS) patients and those related to their rehabilitation. ALAMEDA is an observational study, seeking to explore and identify the most promising means of integrating two principal sources of data: (i) general health and lifestyle retrospective data (as quantified by standard evaluation in the medical domain), and (ii) new streams of patient monitoring data.

The latter, in its turn, uses two modalities of data collection: (i) automatically obtained, objective data, gathered through the use of wearable devices, and (ii) subjective, self-reported patient outcomes (PRO) retrieved through the use of mobile applications. Together, the two modalities cover data that look at the everyday activities, sleep behaviors, emotional status and quality-of-life (QoL) aspects of a patient.

Studies similar to the one proposed in the ALAMEDA project have been conducted previously. They have focused both on long-term PRO monitoring (e.g., PD_Manager [10], mPower [11], MO-BITEC [12], PROMOPROMS [13]) as well as on specific motor impairment analysis (e.g., unstable walking patterns and fatigue in MS [14], analysis of ambulation data from recovering stroke patients [15], and tremor detection for PD [16,17]). These related studies, as well as others, are analyzed in more detail in Section 3.1. However, it is worth noting that ALAMEDA distinguishes itself from such previous studies through the following aspects. The proposed wearable devices (see Section 2.2.1) are minimally intrusive and work in tandem with one another such that their data output and their positioning on the body (soles, hip and wrist) cover the information needed for useful predictions highlighted in previous studies at a lesser burden for the patient. Furthermore, the ALAMEDA study is designed to offer two overlapping data collection directions: the continuous PRO-based data collection, which runs for one year, and the quarterly 1–2 week-long milestones of intense monitoring, where wearable data are collected under both clinical, as well as free-living conditions (see details in Section 2.1). While ALAMEDA has a lower number of patients per study, the diversity and amount of data per patient make it both novel and suitable for the exploration of the most promising health status prediction setups using machine learning (ML)-based approaches.

Considering the project context, it is worth noting that it has a significant exploratory role. The overall objective of the PD, MS and stroke studies is to investigate the potential of the outlined data streams to predict outcomes of disease-specific standard medical tests (or a change in these outcomes from one milestone to another) and to validate the feasibility of the proposed monitoring protocol to be extended to follow-up studies with larger participation. Study-specific objectives are further specified when introducing each pilot use case.

Given these objectives, the collected data are subjected to an AI/ML-based analysis aiming to uncover which prediction setups (combination of input data sources and health status targets) are feasible under the proposed data collection protocol. We aim to examine prediction setups that are both longitudinal (predict change in health status target from one milestone evaluation to the next) and cross-sectional (predict momentary health status target based on a history of multi-modal inputs).

The result of this analysis is intended to guide clinicians in the design of data collection procedures for future, large-scale trials that (i) can validate the generalization potential prediction setups identified in ALAMEDA and that (ii) can evaluate the usefulness of modifications to pharmacological and non-pharmacological therapeutic options (e.g., different exercise regimens) that are informed by these AI/ML predictions.

To respond to the mentioned objectives, ALAMEDA adopts the guiding principals of digital transformation [18] applied to organizational activities, health assessment processes and models to leverage the opportunities of the proposed mix of digital technologies. It is the scope of this article to describe the exact setup of the digital transformation process for each of the three considered brain diseases, as well as to present the methods by which we evaluate the feasibility of the process and the utility of the proposed prediction setups.

### ALAMEDA Use Cases

Three major brain diseases and corresponding pilot sites are targeted in ALAMEDA: (a) the PD pilot study in Greece, at the sensing Movement Disorders Clinic of the First Department of Neurology at Eginition Hospital, Athens University Medical School (NKUA); (b) the MS pilot study by the Italian MS Foundation (FISM, the leading funding agency of research in Italy and the third worldwide in MS field) and its society (AISM, which relies on the AISM Rehabilitation Service Ligure in Genoa, Italy); and (c) the stroke rehabilitation pilot study at the Neurology Department of the University Emergency Hospital of Bucharest (UHB), Romania. All three pilot studies share a main approach to evaluate the success of the digital transformation process: (i) determine the feasibility of the data collection protocol, judged by the adherence of patients to it and their experience in interacting with the applications and devices, and (ii) analyze the ability of the digital data to reflect on the patients’ condition as quantified by the accuracy of AI models that are developed for the prediction setups specific to each disease (see more in Section 2.4). However, each study also aims to explore a separate set of characteristic research interests.

The PD pilot implements a clinical study to assess the use of sensors in monitoring the motor and non-motor aspects of advanced Parkinson’s disease. The care of patients with advanced Parkinson’s disease (PD) is complicated, as both motor and non-motor manifestations of the disease worsen over time and seriously impair the quality of life (QoL) of patients and their caregivers. Various scales have been devised to monitor the motor and non-motor aspects of the disease, but they are subjective in nature, subject to recall biases, they do not have good temporal resolution, and are not easily quantifiable. In contrast, technology-based applications to monitor various aspects of the disease have the potential to provide objective quantifiable data over a long period of time, and to capture more accurately the temporal fluctuations of the disease phenomena [19]. In particular regarding sleep, which represents a serious problem for many patients with PD, there is a need to be able to monitor sleep in patients with PD with an easily applicable device in the home environment.

The primary goal of the PD pilot is to explore the capability of detecting meaningful worsening of the global status or of the individual motor or non-motor aspects of Parkinson’s disease. This is performed by investigating the use of PRO and wearables-based data in combinations that can predict the measurements in classical PD scales, such as the MDS-UPDRS and QoL-related measures, the MoCA cognition scale (members of each medical partner undertaking a pilot study—NKUA, FISM and UHB—have completed the appropriate training required for use of the MoCA test) and anxiety/depression scales. A complementary objective is to correlate simple device recording during sleep to specific PD-related sleep scales and to polysomnographic recordings.

The MS pilot engages in the line of rehabilitation research, conducted by the Italian MS Foundation, and focuses on key aspects such as the use of predictive systems to improve decision support systems for multiple sclerosis and the use of wearable technology (from sensors to electronic patient reported outcomes) in MS. The end goal of the MS pilot study is to test AI/ML-based algorithms that are able to predict the risk of developing a relapse in multiple sclerosis. Relapses are one of the cardinal features of MS. Relapses are the unprovoked and unforeseen temporary worsening of physical disability, sometimes causing permanent severe disability [20]. Relapses are extremely important for the QoL of PwMS and have a major influence on treatment decisions [21]. However, their timely diagnosis is often difficult.

Therefore, a characteristic research interest of the MS study is to explore the use of combined PRO and wearable-provided data as input for relapse prediction algorithms.

The stroke rehabilitation pilot aims to use the data modalities available in ALAMEDA (see Section 2.3) to complement and extend the information obtained through typical processes applied to chronic patients that have suffered a stroke. The follow-up on the cognitive and motor rehabilitation process of stroke patients is an important activity towards ensuring that patients (especially chronic ones) can make steady steps towards regaining their autonomy and improve their quality of life. Due to a high number of patients with stroke-related motor and neurocognitive deficits [22], the long-term accessibility of this group of patients to rehabilitation centers remains low, even in well-developed countries. Thus, the purpose of the extended monitoring implemented in ALAMEDA is to allow physicians to have a continuous update on the patient rehabilitation process in between clinical visits.

The characteristic research interest of the stroke pilot is to explore the support provided by AI/ML algorithms to distinguish between different levels of severity for movement and cognitive impairment as quantified by standard neurological tests (see more in Section 2.4). A complementary objective is to develop a ML approach to detect the execution of rehabilitation exercises based on input from wearable devices (notably, accelerometer-based, wrist-worn bracelets).

The previous considerations motivate the study protocols and data collection journeys that we detail in the sections to follow.

## 2. Materials and Methods

The ALAMEDA Pilot studies are 1-year longitudinal observational studies in PD, MS and stroke that are part of the ALAMEDA project, financed from the European Union Horizon 2020 research and innovation program, under grant agreement No. 101017558. One of the central objectives in the project is to design a continuous digital transformation methodology that makes the process of continuous, remote monitoring using wearable devices and mobile applications for patient reported outcome and experience submissions operational. A specific goal is also to assess the feasibility of data collection by monitoring participants’ adherence to the scheduled requests and usability of the toolkit by means of validated questionnaires. The type of registered data and the process by which they are collected are conceived so as to closely fit the particularities of Parkinson’s disease, multiple sclerosis and stroke care necessities.

In this section, we start by defining the characteristics of each pilot study in terms of spatio-temporal coordinates, number of participants and their inclusion and exclusion criteria, as well as the main objective of prediction (see Section 2.1). We then detail the data collection journey, describing the set of devices and applications used for registering data (Section 2.2), and determining the exact content and schedule of data collection proper to each use case (PD, MS and stroke—Section 2.3). The list of intermediate and final prediction objectives, as well as conditions for raising alerts over collected data, are detailed in Section 2.4.

### 2.1. ALAMEDA Pilot Studies

The ALAMEDA project focuses on pilot studies for Parkinson’s disease (PD), multiple sclerosis (MS) and rehabilitation after stroke. All the studies are observational in nature, meaning that no direct intervention in the typical medical treatment protocol for each of the mentioned diseases is performed during the pilots. The study duration per patient is of 1 year, and each pilot takes place between June 2022 and September 2023.

It is worth noting that the project has a significant exploratory role. It is focused on research into identifying and designing a novel process of data collection that involves combinations of PRO and wearable device based data streams. We subsequently investigate the potential of these data streams to predict outcomes of disease-specific standard medical tests (or a change in these outcomes from one milestone to another). Under these considerations, the temporal (one year for the actual monitoring of all patients) and human resource constraints of the project reflect themselves in the number of patients that can actively be enrolled in the pilot studies.

In what follows, we present the typical condition of patients for each disease that will lead to their inclusion in the observational studies.

#### 2.1.1. Parkinson’s Disease

The typical subject for the present observational study is a middle-to-older aged person, either male or female, with a proven diagnosis of advanced hereditary Parkinson’s disease. The range of persona selected for the pilot includes people with different habits but with the ability to perform the various foreseen exercises and tasks.

A total of 15 participants are selected from the group of patients with advanced PD, who are followed at the Special Outpatient Clinic of Parkinson’s Disease and Related Movement Disorders at the First Department of Neurology of the National and Kapodistrian University of Athens (NKUA), at Eginitio Hospital. Inclusion/exclusion criteria are detailed in Table 1. The availability of a caregiver who can assist, if needed, in aspects of the study is desirable but not necessary. There are no restrictions in patient selection regarding race, ethnicity or sex, but the age group is circumscribed (30 to 75) so as to be, on the one hand, somewhat representative of the PD population at large and, on the other, avoid comorbidities associated with aging that may overshadow the effects of PD itself. Note that the advanced PD stage refers to that time over the disease course when motor and non-motor fluctuations and dyskinesia are encountered [23]. The H&Y 2.5 or less at the “on” phase is chosen since the ALAMEDA PD pilot requires advanced PD patients with fluctuations to monitor with sensors. The exploratory nature of the PD pilot, requiring PRO submission and wearing of devices, leads us to exclude the confounders (including the age group of 75, which is not familial with the use of sensors). A MoCA score of 25 higher is generally considered normal, while a score of 18 to 25 can indicate mild cognitive impairment, and 10 to 17 can indicate moderate impairment. A score of less than 10 indicates severe impairment [24]. A diagnosis of PD with dementia is made upon the MDS recommendations [25]. Study participants are asked to return for re-evaluation to the clinic at 3-month intervals, because 3 months is the interval that is common in the NKUA movement disorder clinic where PD patients are examined.

#### 2.1.2. Multiple Sclerosis

The typical subject for the MS observational study is a young-to-middle age person, either male or female, with a proven diagnosis of a relapsing–remitting form of MS. Participants included in the pilot may present different levels of disability (from minimal to mild) and different habits. However, all of them are able to perform the task foreseen by the MS use case either independently or with minimal support. The study enrolls 20 subjects with MS who receive care in one of the rehabilitation centers managed by the Italian Multiple Sclerosis Society. The key inclusion and exclusion criteria for participation in the study are presented in Table 2. We note that MS can strike at any age but is typically identified between the ages of 20 and 30 [27]. Relapsing/remitting multiple sclerosis (RR-MS) makes up 85% of MS cases [28]. As for natural history, after 15 years, about 60% RR-MS patients will convert to secondary progressive MS (SP-MS) [29]. However, known biomarkers of disease activity are currently less useful in detecting the transition from RR to the SP form [30]. Therefore, to maximize the possibility of monitoring health parameters from participants in an active phase of MS, we consider as exclusion criteria an age greater than 30+15=45 years. Concerning the other inclusion criteria, we focus on patients with low disability (here, EDSS < 4.5) since the use of wearable technology has been validated in this population (see Yousef at al., 2017 [31] for a review). The patients are followed up with for 1 year after ALAMEDA participation in order to register any relapse in this period (making the total monitoring time 2 years). This is used to check whether any of the employed ALAMEDA data sources (see Section 2.2) are predictive of the event.

#### 2.1.3. Stroke Rehabilitation

Fifteen stroke patients that are engaged in the stroke rehabilitation process are enrolled at the Department of Neurology of the University Emergency Hospital of Bucharest. The criteria for inclusion or exclusion in the study are presented in Table 3. It is to be mentioned that patients with severe neurological deficits are excluded from the study, as this type of patient is not able to use the devices proposed by the ALAMEDA project.

The typical subject for rehabilitation after the stroke pilot study is an adult falling within a wide age group (18–85) but with a distribution skewed heavily toward those over 50 years in age, as the prevalence of stroke increases with age; however, about 25% of ischemic strokes occur in middle-aged patients who are still working [32]. The subject has suffered a stroke, whereby their motor, balance, gait or neurocognitive abilities are impaired. Stroke survivors face a long-time chronic condition, while rehabilitation is usually started during hospitalization and continued for at least 6 months in a neuro-rehabilitation facility or at home, usually under the guidance of a specialist. This period is considered the most important for neuroplasticity after stroke, especially the first weeks after the event [33], which is why we chose to enroll patients that were hospitalized for stroke in the last month. Most stroke patients experience muscle weakness of the upper or lower limbs. Additionally, difficulty in walking and maintaining balance are two of the most devastating sequelae of a stroke, and the restoration of gait is often one of the primary goals of rehabilitation.

#### 2.1.4. End-User Engagement

As digital technology continues to evolve rapidly and healthcare providers and ACT policymakers work hard to adapt, there is a high risk that the patient perspective may be lost. Thus, meaningful patient engagement is set at the top of the priorities of ALAMEDA, where the learnings of previous projects and experiences are considered to ensure it is carried out in the most effective, participatory and purposeful way. Specifically, ALAMEDA relies upon the participation of the Italian Multiple Sclerosis Society Foundation (FISM) as a key partner and former coordinator of the MULTI-ACT project (https://www.multiact.eu/, accessed on 11 August 2023), exploiting the gained knowledge and guidelines to build a strong end-user (and specifically) patient-engagement route along the whole project’s duration. To this end, within ALAMEDA, two types of steering and consulting committees are established (please refer to public project’s deliverables (https://alamedaproject.eu/public-deliverable/, accessed on 11 August 2023) D7.1 and D7.3): the Engagement Coordination Committee (ECT) and three local community groups (LCGs) in the respective countries, i.e., Italy (multiple sclerosis), Greece (Parkinson’s disease) and Romania (stroke). LCGs are composed by 9–15 end users and animated by the respective patients and clinicians sitting in the ECT so as to secure engagement at the national and disease-specific levels and provide valuable feedback as the research work progress. The aforementioned committees have also participated proactively in the design phase by providing their preferences with regard to the data collection modalities and frequency, as well as the use of the proposed wearable and sensors (as they are presented next). They are continuously actively engaged to support the research team throughout the project concerning the next steps in order to elevate its acceptability and usefulness.

### 2.2. ALAMEDA Data Collection Tools

To implement the observational studies described previously, a set of wearable devices are employed, and novel customized mobile applications are developed to enable the underlying data collection. The list of devices and software applications is presented in this section, while a mapping of their capabilities to the type of variables of interest for each pilot study follows in Section 2.3.

#### 2.2.1. ALAMEDA Wearable Devices

**Figure 1:**
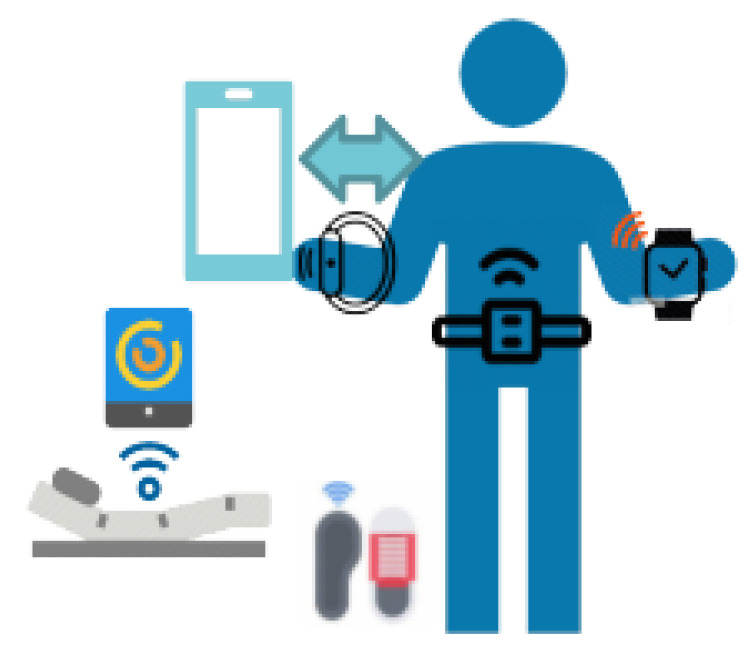
A schematic of the set of wearable devices used in ALAMEDA pilot studies: smart watch, accelerometer bracelet, IMU-sensor belt, ground force sensitive insoles, smart mattress. The figure shows the points on the human body where each device will be worn.

The set of devices to be used in ALAMEDA pilots covers the retrieval of information from two main health status domains: general activity/motor function and sleep. A schematic of the type of considered devices is shown in Figure 1. Apart from the health information domains they cover, the devices can be further classified by two criteria: (i) continuous vs. limited duration usage, and (ii) commercial vs. experimental development. Their complete list, their usage mode, and the extracted metrics are summarized in Table 4 and described more closely in what follows.

##### Smartwatch

The participants are given a Fitbit Versa series (https://www.fitbit.com/global/eu/products/smartwatches/versa-lite (accessed on 11 August 2023), https://www.fitbit.com/global/us/products/smartwatches/versa4 (accessed on 11 August 2023), https://www.fitbit.com/global/us/products/smartwatches/versa (accessed on 11 August 2023)) smartwatch to wear continuously throughout the study. From the smartwatch, we collect objective general information about (i) physical activity levels during the day (number of steps, distance traveled, calories burned, and minutes spent per physical activity level—inactive, light, moderate and vigorous), and (ii) sleep information (minutes spent in each sleep stage: light, deep, and REM—sleep efficiency), and (iii) general health condition—heart rate statistics and oxygen levels. The literature has shown that Fitbit devices provide a valid estimation of the number of steps in both laboratory [34,35,36] and free-living conditions [37,38,39], with accuracy between 0.9 and 1.0.

##### Smart Bracelet

Participants will wear an ActiveInsights GENEActiv bracelet (https://www.activinsights.com/technology/geneactiv/, accessed on 11 August 2023) during a period of intense monitoring (see more in Section 2.3. The GENEActiv has a triaxial accelerometer with sensitivity in the −8–+8 g range, skin temperature and luminosity sensors, and has the capability to record data continuously for 15 days at a 50 Hz sample rate. The device is specialized for activity and sleep tracking and has been used in over one hundred clinical trials or observational studies [40]. Some extractable metrics overlap with the Fitbit capabilities (e.g., number of steps, minutes spent in different physical activity levels, and sleep efficiency), but one key difference is the access to raw accelerometer data, which enables the application of custom ML algorithms for the detection of (i) tremor, dyskinesia or hypokinesia episodes in PD patients, (ii) the execution of physical rehabilitation exercises in stroke patients or (iii) the smoothness of upper limb movements, stumbles or falls in MS patients. An additional benefit of the sensor is the ability to be attached to other body parts (e.g., waist or ankle) apart from the wrist.

##### Smart Insoles

During the defined period of intense monitoring, patients are asked to wear a pair of Loadsol-AP insoles (https://www.novel.de/products/loadsol/, accessed on 11 August 2023), developed by Novel Gmbh, which are capable of sensing the ground reaction force exerted at two distinct areas of the foot, the heel and the forefoot. Measurements are sent with a frequency of up to 200 Hz, over a Bluetooth connection, to the user’s smartphone. These insoles are used to study walking and running gaits in real-world settings [41,42]. In the ALAMEDA pilot studies, the insoles can readily provide accurate measurements related to gait metrics, such as step number, cadence, step cycle time, loading rate, factor of imbalance (disproportionate loading of one foot compared to the other), or peak push force. Furthermore, analysis of the raw data can reveal the degree of balance problems or the severity of a walking defect/gait instability, which is of relevance specifically in the stroke study, or even identify patterns indicating improvement or not during the rehabilitation sessions.

##### Smart Belt

This is a prototype wearable device developed by a member of the project consortium (Norwegian University of Science and Technology—NTNU) to record patient gait and physical activity data. The belt is composed of three motion tracking sensors mounted on a belt (one at the bottom of the spine and two on the sides of the waist). Each sensor is built using an inertial measurement unit (IMU) to record the linear and angular acceleration of the body and a Wi-Fi-based communication system to send data to a cloud platform (the ALAMEDA Research Data Management Platform). The sensors have a continuous operation lifetime of 20 h after a full recharge and have the ability to store the collected data locally for up to 30 days. The sensors are worn by the patients during the intense monitoring period to collect information in order to complement the data from the insoles in terms of specific basic activities (e.g., walking vs. standing or lying), as well as gait or balance issues.

##### Under-Mattress Sensor

Patients will use a commercial, CE certified, under-mattress pneumatic sensor (Withings Sleep Mat (https://www.withings.com/ro/en/sleep-analyzer, accessed on 11 August 2023)) to obtain more accurate and complementary data for the analysis of their sleep cycles in a completely unobtrusive manner. The device performs continuous heart rate measurement while sleeping and has embedded audio sensors that help detect sleep apnea and snoring using proprietary algorithms. The Withings sleep mat is easy to set up and can operate continuously once plugged in. The collected sleep data are highly relevant for all three pilot studies but especially for the PD case, where additional properties for the identification of sleep disorders are under investigation in collaboration with the sleep clinic of Attikon Hospital in Athens.

##### Mattress Topper Pressure Sensor

This is a prototype device developed by a member of the project consortium (ENORA Innovation) and intended for the advanced monitoring of sleep. The device will be used at the premises of Attikon Hospital (Greece) in the sleep clinic, where participants of the PD use case will also undergo a sleep study at the intense monitoring periods and upon their enrollment. This procedure is for evaluation purposes since it is a prototype device and if successful, then it will also be available for the remaining studies of MS and stroke. The mattress topper is built of a flexible conductive fabric and pressure sensitive plastic (velostat) displayed in a 16 × 16 grid, which can be laid over a mattress, underneath the bed sheet. The prototype device records a heatmap of body postures at five frames per minute and has additional sensors for monitoring the sleep environment (sound level, light level and temperature). The device is used in correlation with a polysomnography analysis to annotate sleep disturbances and investigate the predictive power of sleep position and environment condition variables on the quality of sleep.

#### 2.2.2. Applications for Patient Reported Outcome Collection

In the ALAMEDA Project, a suite of mobile applications enables the interaction of the patients with the ALAMEDA platform to submit patient reported outcomes (PROs) and receive notifications and alerts, as well as visualize and keep track of their current health status with regards to various aspects (e.g., sleep, gait, mobility, and psychological). Collectively, the developed applications form the ALAMEDA Digital Companion. The functionality of the components is briefly described in what follows.

**The WellMojo application** is the central Digital Companion App interface and the application that is responsible for the integration of the rest of the applications in the ALAMEDA platform. WellMojo is the main mobile user interface for the target users, providing daily support in terms of assessing their health status and enabling them to self-manage their condition through coaching and tips. WellMojo provides a dashboard allowing for the presentation of patient related data in the nutrition, sleep, social, mood and physical activity aspects (including also details and historical data), as well as presentation and management of (standard medical and pilot study specific) questionnaires and their answers. The applications offers push notifications acting as reminders for taking actions or for presenting PMSS-related information. Moreover, WellMojo implements a very simple annotation interface, by which patients can mark intervals and timestamps of executing certain activities (e.g., physical rehabilitation exercises), experiencing symptoms (e.g., tremor and stumbling), or medication intake. In ALAMEDA, medication affecting the symptoms of the disease is considered (e.g., levodopa—L-DOPA [43] in PD) in order to, apart from keeping a diary, also provide information about the measurements from the wearable sensors.

**The ALAMEDA Conversational Agent** is responsible for implementing an alternate means of PROs collection, especially for questionnaires which are complex in nature (e.g., where the answer to one question influences the kind of questions asked next) and which are suitably modeled as conversations. Additionally, a particular focus is given to questions where patients respond regarding their emotional well-being, perception of social support and quality of life. To ensure a unified way to interpret and quantify the data collected from all the participants, predefined options (e.g., as buttons) are presented to the user for all standardized questions prepared by medical professionals. To take advantage of the conversational nature that a chatbot offers and make the interactions more personal, data in a free-text form are also collected. A notification system is developed to maintain user engagement and ensure that the established questionnaire submission schedule is respected. Additionally, follow-up notifications are sent as reminders, in case some questions were left unanswered.

**The mood estimation android application (MEAA)** is responsible for monitoring the user facial expressions and estimating his/her mood accordingly. The application was developed as a service and is integrated with other components of the ALAMEDA Digital Companion. MEAA runs in the background, minimizing distraction on behalf of the user, and is active only while the user is actively engaging with WellMojo or the ALAMEDA Conversational Agent. The service engages the front camera of the smartphone to receive incoming frames, which are analyzed locally by the application to classify the facial expression as one of “angry”, “disgust”, “fear”, “happy”, “sad”, “surprise” or “neutral” categories.

**The virtual keyboard** is designed to assess the typing patterns of a user on a mobile device. It runs as an installed keyboard service on an Android smartphone and works similarly to the Android OS default keyboard, including all modern functionalities, such as word prediction and auto-correction. The virtual keyboard application is designed to unobtrusively record and analyze typing patterns of smartphone users and relate them to certain conditions, such as depressive states.

Apart from the above-mentioned applications installed on the user’s smartphone, ALAMEDA features two tablet applications, both developed within the consortium of ALAMEDA, which are used for cognitive and motor ability evaluation at the specific milestones defined by the medical partners of the project during the pilot studies.

**The virtual supermarket test application (VST)** is an app designed to assess older adults’ cognition through a simple task modeled on an everyday activity. The application aims at activating a multitude of cognitive processes namely visual and verbal memory, executive function, attention and spatial navigation, with the emphasis placed on the executive functionality. The latest version of the VST [44] includes advanced navigation metrics with the virtual space divided into three zones (green, yellow and red). Different zones represent different deviations from a pattern of optimal navigation for task completion. The diagnostic utility of the VST has been validated in different populations and it has also been validated against electroencephalography (EEG) biomarkers.

**The line-tracking test application** is designed to assess older adults’ hand dexterity. Developed within the NoTremor EU project (https://cordis.europa.eu/project/id/610391, accessed on 11 August 2023), the line-tracking test measures the ability to follow a randomly moving target (the cyan line) while ignoring the distracting target (the red line). The line-tracking test can identify different components of the hand movement (e.g., reaction time, movement time, and several internal time delays).

#### 2.2.3. ALAMEDA Data Collection Conceptual Architecture

To enable the collection of data from the previously mentioned device and software application sources, dedicated and integrated information flows are set up.

**Figure 2:**
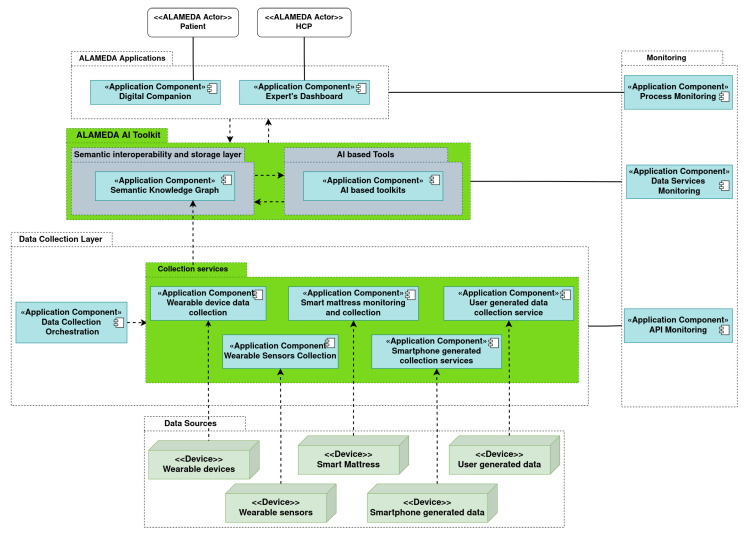
A component-wise overview of the ALAMEDA data collection architecture outlining the relevant information flows and visualization methods.

Figure 2 shows the component-wise overview of the data collection architecture. There are dedicated services for each wearable device, which operate either automatically (e.g., for the Fitbit smartwatch) or on demand (e.g., data collection for the GENEActiv bracelet or the Loadsol insoles). Separate collection services are set up for the software applications composing the ALAMEDA Digital Companion used for PROs—the WellMojo application and the Conversational Agent interface. All questionnaire (see Section 2.3) and relevant question answers (see Appendix A Table A1, Table A2 and Table A3) submit data to the ALAMEDA semantic knowledge graph (SemKG), which uses a custom-designed ontology for the vocabulary of collected data modalities. The SemKG also receives aggregate values (e.g., step counts, activity levels, sleep stage durations, and average load balance) from the data collection processes that manage the smart devices. The processing and evaluation of data (e.g., to detect dyskinesia or tremor events, to detect rehabilitation exercise sessions, and to classify the result of a medical test during milestone evaluations) is performed using a set of AI tools that are accessible as RESTful API services. Data from the SemKG, as well as the results obtained from the AI toolkit can be inspected in a browser-based UI called the expert’s dashboard, where medical professionals can view the collected data in a table form, as well as in a graphic manner.

**Figure 3:**
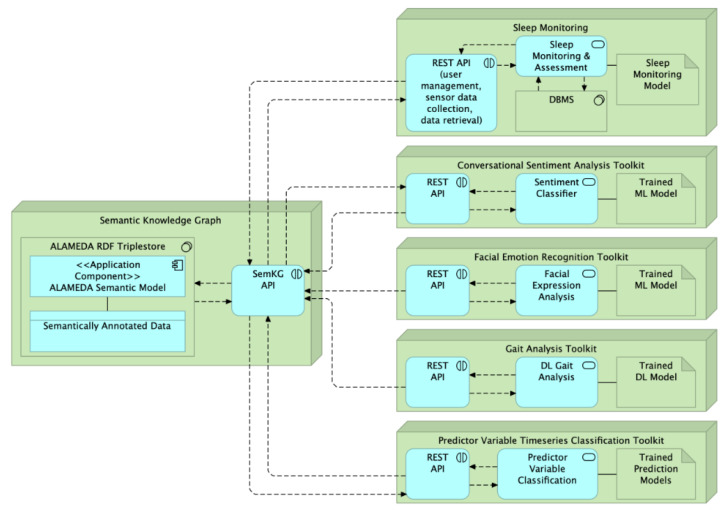
Overview of the ALAMEDA AI Toolkit services and their interdependence. Dotted lines in the figure indicate direction of communication between the toolkit services. For example, the Predictor Variable Timeseries Classification Toolkit receives input from all the other toolkits (sleep assessments, motor symptom or activity summary aggregates from the Gait Analysis toolkit, etc.) through the SemKG API and will in return store its prediction results for patient health status in the Semantic Knowledge Graph.

The components of the ALAMEDAA AI toolkit are shown in Figure 3. Note again the central role of the SemKG service as a repository for the input (PRO data or aggregate metrics from the smart devices), as well as the prediction output of AI services. Data annotation occurs in two manners. During milestone clinical visits (see Section 2.3), medical professionals manually annotate tests with the name, timestamp and result of the medical test or exercise session. Patients can also annotate manifestations of their disease (e.g., tremor or dyskinesia events, and stumbling), medication intake (especially for PD patients), or the execution of exercise sessions (e.g., rehabilitation exercises for stroke patients) using a simple, single-button mobile interface with predefined options per pilot study, which is part of the ALAMEDA Digital Companion.

Regarding the available AI services, two of them focus on a multi-modal evaluation of the patient’s emotional status using conversational and facial expression input. One service is dedicated to sleep monitoring, providing analyses, which are further detailed in Section 3.3. The Gait Analysis Toolkit encompasses a larger set of predictions for motor impairment detection and exercise session detection based on input from wearable devices, such as the NTNU smart belt, the GENEActiv bracelet or the Loadsol insoles (see detection targets in Table 5). The Predictor Variable Time Series Classification Service is the workhorse service hosting the set of models developed to make predictions of the patient health status in various longitudinal and cross-sectional prediction setups. The possible targets are listed in Table A4, Table A5 and Table A6 in Appendix B, while the model development is detailed in Section 3.2.

### 2.3. ALAMEDA Data Collection Journey

The ALAMEDA data collection journey refers to the experience that a patient enrolled in an ALAMEDA pilot study will have in terms of the exact variables of information that will be collected from them using the devices and software applications presented in Section 2.2, the schedule of interacting with these and the existence of special activities to be performed in order to better assess disease and non-disease related factors, which influence living with the PD, MS or stroke. The aforementioned “journey” was defined with the help of the clinical partners of the project and customized to the needs and requirements posed by each use case individually.

For each study in particular, we highlight (i) the list of information variables we collect and the means by which this is performed, and (ii) the data collection schedule, distinguished into continuously monitored parameters and PROs and intense (special) monitoring periods. The latter is formed as a data collection protocol summarized in Table 6.

The collected variables are organized into five health status categories: (i) mobility, general motor or physical function; (ii) sleep disorders; (iii) mental and cognitive ability; (iv) emotional status; and (v) quality of life and daily living.

Table 6 shows a phase that operates continuously throughout the pilot to collect data and a phase that calls for more intense monitoring but of limited duration, using additional devices devices and requests for activities from the patients. The intense monitoring is limited to a maximum of two weeks at every milestone (as set by each pilot) in order to reduce the patient load and to become accustomed to the recording and charging limitations of some devices (e.g., the GENEActiv bracelet can record data at 50 Hz for up to two weeks before a recharge and reconfiguration are needed). It is worth noting that the specific duration of the intense monitoring period for each pilot study is the result of a combination of two factors, the activity and monitoring protocol proposed by each study (see following subsections), and the preferences of patients as resulting from the research study co-design sessions carried out by the local community groups (LCGs) (cf. Section 2.1.4), which expressed, among others, the likelihood of adherence to the intense monitoring requirements as a function of duration. An example of a difference between the pilot studies is the preference of PD patients for an intense monitoring period of one week, as opposed to the two weeks preferred by those with MS and stroke.

#### 2.3.1. Study Design for PD Pilot

The Parkinson’s disease pilot study will enroll 15 patients with advanced PD who are followed at the Special Outpatient Clinic of Parkinson’s disease and Related Movement Disorders at the First Department of Neurology of the National and Kapodistrian University of Athens (NKUA), at Eginitio Hospital. The study considers four milestones in-clinic evaluations at 3-, 6-, 9- and 12-month time marks. Each milestone is preceded by a 1 week intense monitoring period.

For the Parkinson’s disease study, the set of variables to be monitored are presented in Table 7. For each variable, the name, possible range of values, and acquisition method (PROs input application or set of devices) are indicated, as well as the type of the time period of collection, being either continuous or of limited duration.

Apart from the set of the standard medical questionnaires considered, the PROs of PD patients also consist of a number of relevant questions, which inform of the subjectively rated patient experience of “on”/“off” states, the severity of dyskenisias and other motor complications, the emotional state and the degree of social interaction. These questions are scheduled to be answered weekly, but the patient can complete a question session over the course of several days in the week. The full list of questions and their scheduling is displayed in Table A1 included in Appendix A.

During the intense monitoring period, the patients follow a protocol to ensure reliable data and patient adherence. They are given the smart bracelet for one week to wear at home. The device does not require any interaction by the user (no added burden). The MDS-UPDRS is performed at the first visit, once when the patient is in the on phase and once when they are in the off phase. PD patients are instructed to install and calibrate the Withings mattress at home. Clinicians monitor adherence from the Heath Mate App and may adopt some strategy to increase it (e.g., messages to remind of mattress use, phone call to explain again the installation and calibration procedure, and site visits if needed). Participants are asked to wear the smart insoles and the smart belt and perform a set of predefined walking tasks during the visit monitoring day. Patients are instructed and reminded to keep a simple diary of activities performed during the day (selecting from pre-defined options) using the WellMojo application.

#### 2.3.2. Study Design for MS Pilot

The multiple sclerosis pilot study will enroll 20 patients who receive care and counseling in rehabilitation centers of the Italian Multiple Sclerosis Society, most notably near the city of Genoa. The study considers milestones of in-clinic evaluations at the 6, 9 and 12 month time marks, whereby each milestone is preceded by a 2-week long intense monitoring period. The list of monitored variables, and the acquisition method and frequency, as well as the used devices, are shown in Table 8.

Similar to the PD pilot, for the MS study, a list of additional non-standard, subjective experience relevant questions is considered as PROs. In the MS case, the relevant questions cover all the five health categories, and their main purpose is to ascertain symptoms and situations experienced by the patients which might be indicative of early-stage disease relapse. Relevant questions are delivered on a daily basis, but the patient has the option to postpone the answer for up to one day. The full set of questions and their frequency is summarized in Table A2, included in Appendix A.

The intense monitoring period for the MS study instructs patients as follows. Participants wear the smart bracelet for two weeks (24/7). The device does not require any interaction by the user (no added burden). They are instructed to install and calibrate the Withings mattress at home. Clinicians monitor adherence from the Heath Mate App and may adopt some strategy to increase it. Patients are asked to wear the smart insoles about 30 min/day in an active phase of the day. Suggested slots are the way from home to work (and/or way back), leisure-time walks, daily-life activities at home, and exercise/rehabilitation. Participants are asked to complete a diary (either digital through WellMojo App or in paper format) indicating the time of the day they wore the insoles and the activity they performed, selecting the answer in a multiple choice menu. The smart belt is worn during one significant day of the intense monitoring period. Patients select their significant day as one where they perform a motor activity more intensely (e.g., rehabilitation exercise session, and leisure-time physical activity). The WellMojo app is used to keep a diary of the activity performed while wearing the belt.

#### 2.3.3. Study Design for Stroke Pilot

The stroke pilot study will enlist 15 patients who have suffered a stroke and follow a rehabilitation program in the Neurology Department of the University Emergency Hospital Bucharest. The study considers two milestone in-clinic evaluations along the way at the 6- and 12-month time marks, each preceded by a 2-week long intense monitoring period. In addition, because patients are usually hospitalized for a duration of 7–10 days after a stroke incident, the stroke study employs a baseline evaluation at month 0, which serves as reference for the rest of the evaluations during the 1-year pilot study. During their hospitalization, the patients are asked to wear the devices intended for the intense monitoring period every time they perform a standard medical test or a physical rehabilitation exercise. The set of variables to be monitored and the monitoring means and frequency are presented in Table 9. It should be noted that, for the stroke case in particular, there is an explicit entry for detecting whether physical rehabilitation exercises are performed during the intense monitoring period.

All PROs in the stroke study can be completed in one or more sessions within a week. As in the case of the MS and PD pilots, standard PROs are completed by a set of additional relevant questions, whereby for the stroke case, the focus is on the perception of the emotional status and the degree of socializing. The questions and their scheduling are reported in Table A3 in Appendix A.

During the intense monitoring period, patients are admitted to the neurology department of University Hospital Bucharest in order to reduce the burden related to the installation and use of the devices. Participants wear the smart bracelet for the duration of the two weeks since it does not require any interaction by the user. Most of the participants will use the Withings mattress during the intense monitoring period. Patients are shown how to install and use the mattress and can opt to continue using it at home, after the end of the intense monitoring period. The smart insoles and the smart belt are used by patients when they perform their daily rehabilitation exercise. The WellMojo app is used to keep a diary of the daily activities and rehabilitation exercise sessions.

### 2.4. Pilot Study Prediction Outcomes and Alert Triggers

The data collection journey described in the previous section for each pilot study gathers information that is to be used towards achieving the fundamental research goals of each study. The ALAMEDA pilot studies are observational in nature, meaning that the objective is to analyze the predictive power of the collected data, in terms of assessing the patient health status over different, continuous and large time periods. All the while, simple yet informative rules can be defined to make use of the information that is continuously collected (PROs and smartwatch data) so as to highlight situations to which clinicians monitoring the studies should pay closer attention.

From the descriptions in Section 2.3, it is obvious that the analysis algorithms have to contend with streams of data collected over different time horizons (continuously or only during the intensive monitoring period), having varying frequency and different types (numeric, ordinal or categorical). The following subsections detail how each source of data is analyzed individually or in correlation with the others, as well as the conditions that trigger alerts.

#### 2.4.1. Analysis of Data from Wearables

Data retrieved from wearable devices describe in raw measurements movement; thus, each pilot study has defined, specific targets that characterize the movement behavior throughout the day. Table 5 presents the prediction interests for wearable data for each study. For PD, the most important predictions are those with respect to dyskinesia or bradykinesia, as well as the freezing of gait. The MS and PD studies are also interested in detecting restless leg syndrome manifestations during sleep, while the stroke study is interested in detecting physical rehabilitation exercise sessions. Annotations for these events are made by doctors in the clinic visits during the intense monitoring periods.

**Table 9:** Stroke pilot data collection journey—list of monitored variables and their method of collection.

Variable	Description	Data Value Range	Acquisition Method	Used Devices
**Domain I—Mobility, general motor or physical function**				
Step count, periods of relative immobility/slowness of movement	Continuously monitored step count and other features of general mobility in daily life	step count: integer (0–15,000) periods of mobility: seconds (0–14,400)	Continuous Monitoring - eHealth device	Fitbit Smart Watch
Heart rate, SpO${}_{2}$ levels	Monitoring of daily heart rate and blood oxygen levels	heart rate: integer (30–200) blood oxygen levels: percentage (0–100)	Continuous Monitoring - eHealth device	Fitbit Smart Watch
Physical Activity Amount	Exact time periods of inactive, light, medium or vigorous activity	seconds (0–14,400)	Intense Monitoring every 6 months - eHealth devices	Fitbit Smart Watch GENEActiv Bracelet
Rehabilitation Exercises	Detect execution of prescribed upper and lower limb physical rehabilitation exercises	seconds (0–3600) duration of detected exercises	Intense Monitoring every 6 months - eHealth devices	Fitbit Smart Watch GENEActiv Bracelet NTNU Smart Belt Loadsol Insoles
6 min walk test	Sub-maximal exercise test assessing walking endurance and aerobic capacity. Participants walk around an indoor perimeter for a total of six minutes.	metrics from first row avg. cadence per 30 s: integer loading rate: N/s (speed of normal force applied to body) factor of imbalance: percentage (disproportion of load between feet) peak force: N (maximum force push while walking)	Intense Monitoring every 6 months - eHealth devices	Fitbit Smart Watch GENEActiv Bracelet NTNU Smart Belt Loadsol Insoles
ACTIVLIM questionnaire	Self-assessed questionnaire to examine both upper and lower limb muscle strength using daily living activities	integer, questionnaire score: −11–+11	Continuous Monitoring - PRO on smartphone (every month)	Smartphone
Dizziness and Balance questionnaire	Self-assessed questionnaire for the balance variable	integer, questionnaire score	Continuous Monitoring - PRO on smartphone (every month)	Smartphone
Self-assessed questionnaire for muscle tone	Self-assessed questionnaire to quantify the muscle tone variable	integer, questionnaire score	Continuous Monitoring - PRO on smartphone (every month)	Smartphone
**Domain II—Sleep disorders**				
Pittsburgh Sleep Quality Index (PSQI)	Self-administered questionnaire to assess sleep patterns	integer, questionnaire score: 0–21	Continuous Monitoring - PRO on smartphone (every month)	Smartphone
General Sleep Patterns	Continuous monitoring of general sleep stage duration using the smart watch sensor	total bed time: hours (0–12) light sleep: hours (0–12) deep sleep: hours (0–12) REM sleep: minutes (0–240) apnea: Boolean (true/false) snoring: minutes (0–240)	Continuous Monitoring - eHealth devices	Fitbit Smart Watch
Intense Sleep Monitoring	Sleep monitoring during pilot milestones using eHealth devices and a polysomnograph	Previous row metrics + polysomnography analysis	Intense Monitoring every 6 months - eHealth devices	ENORA Sleep Mat Withings Sleep Mat Fitbit Smart Watch GENEActiv Bracelet
**Domain III—Mental and cognitive ability**				
Keystroke dynamics	Detailed timing of typing on smartphone	Enum: classes of abnormal typing patterns	Continuous Monitoring - eHealth devices	Smartphone
Line Tracking Test	Self-administered test on tablet to assess various aspects of arm/hand movement	Reaction time: ms Movement time: msec Internal time delays: msec	Intense Monitoring every 6 months - eHealth devices	Tablet
Virtual Supermarket Test	Self-administered test based on a 3D serious game to assess cognitive decline	time to completion: ms (scores above 215,000 ms indicate possible cognitive impairment)	Intense Monitoring every 6 months - eHealth devices	Tablet
**Domain IV—Emotional status**				
Facial Expression Analysis	Estimate Mood using facial expression analysis enabled by MEAA (see Section 2.2.2)	Enum: mood class and probability	Continuous Monitoring - eHealth device	Smartphone
COAST	Self-assessed questionnaire to assess the speech variable	integer: questionnaire score (20–100)	Continuous Monitoring - PRO on smartphone **(every month)**	Smartphone
PHQ-9	monitor the severity of depression and response to the treatment	integer: questionnaire score (0–27)	Continuous Monitoring - PRO on smartphone **(every month)**	Smartphone
**Domain V—Quality of life and daily living**				
MFIS	Assessment of the effects of fatigue in terms of physical, cognitive and psycho-social functioning	integer: questionnaire score (0–84)	Continuous Monitoring - PRO on smartphone (every month)	Smartphone
Food Habits Questionnaire (FH-Q)	Self-report questionnaire measuring food intake habits about typical eating patterns over the past month	integer: questionnaire score (0–18)	Continuous Monitoring - PRO on smartphone (every month)	Smartphone

In the devices column, the set of input modalities which will facilitate the prediction is indicated. Notice that for each device, we indicate its mount position on the body. As described in Section 2.2.1, the GENEActiv smart bracelet and the NTNU Smart Belt allow for the sensors themselves to be mounted on different part of the body using adjustable straps. Consequently, throughout the intense monitoring periods, patients are asked to alter the positioning of the sensors on their body to assess the predictive capability based on different mount points.

Since the pilot studies are retrospective in nature, the word “real-time” appearing in the description column of some prediction targets refers to the development of algorithms which require a small time interval around the start and end timestamps of the events. To facilitate ground-truth annotation collection, patients are instructed to use the intuitive annotation functionality provided by the WellMojo application of the ALAMEDA Digital Companion, which has a predefined list of target events and requires a single button press to mark the occurrence of an event.

#### 2.4.2. Analysis and Alerts on Combined Wearables Data and PROs

As explained in the introduction, the approach taken in the ALAMEDA Pilot studies is an AI/ML-first exploratory analysis of the predictive capability of the proposed data collection journey. We set up the ML objectives as two types of prediction tasks, corresponding to either a cross-sectional or a longitudinal analysis. These can be seen in the tables included in Appendix B: Table A4, Table A5 and Table A6.

For cross-sectional-like prediction setups, each pilot study proposes a multinomial classification target based on thresholding the score of a standard test questionnaire in a manner that is clinically relevant. MDS-UPDRS [45], EDSS [46] and mRS [47] are the most important health status questionnaires for the PD, MS and stroke studies, respectively. Apart from these, classification setups can be made for other medically relevant questionnaires (e.g., MoCA [48]), covering all five health status categories outlined in Section 2.3.

The other type of prediction setup resembles a longitudinal analysis. For the same tests as above, a binary classification task is defined, posing a change vs. no change (or change by X points vs. change by less than X points) in between any two milestone evaluation moments of the study.

The novelty of our approach stands in the development of ML models that can exploit several types of input at the same time to perform the classification tasks mentioned above. Specifically, we employ both the detection results from wearable devices, as well as PRO and physical activity and sleep summaries obtained from the Fitbit smartwatch as input. We explore the prediction capability of momentary snapshots (e.g., data collected only during the intense monitoring period), as well as that of longer-term metrics from PRO and Fitbit data aggregated over the time period from one milestone evaluation to the other.

Though we mentioned that ALAMEDA pilot studies are observational in nature, it is the case that changes in some of the monitored variables (from the Fitbit smartwatch, Withings Sleep Mat, or from received PROs) provide valuable and actionable insights to medical professionals with respect to the health status of the patient. Consequently, a set of alert conditions are designed, which highlight abnormal situations and display them in a dashboard reserved for the monitoring of the pilot study progress. The variables subject to alert conditions and the criteria for triggering them are listed in Table 10. The rule trigger conditions are selected as changes with a low probability of observance in the given monitoring time frame (one year) such that if they are observed, a notification should be raised.

## 3. Discussion

To put the ALAMEDA pilot studies into greater perspective, in what follows, we review the related work, discuss the analysis of the influence of non-disease related factors on the evolution of PMSS patients, and present the expected challenges within the data collection journeys and the means to mitigate them, as well as going over measures that ensure data privacy once the collected information is shared with third-party entities.

### 3.1. Related Studies

The use of wearable sensors and digital methods to collect data about the patient status or progress of the recovery treatment is an ongoing area of research and a field exhibiting high innovation for related applications, with many examples to date (as shown in what follows). However, there is a large variance in the duration, scope and method of the monitoring. Most of the studies involving the use of wearable sensors occur in laboratory environments and are of a limited duration (up to 2 weeks), while most of the long-term studies (e.g., extending to more than 6 months) collecting patient-reported data do not include objective data measured by specialized devices at the same time.

The ALAMEDA pilots are deployed in a one-year study, where digitally completed PROs are accompanied by objective measurements from an unobtrusive device (a smart watch), while more intense monitoring is scheduled at specific milestones of the studies (3-, 6-, 9- or 12-month marks), wherein a novel combination of wearables (the accelerometer bracelet, the IMU sensor belt and the ground force measuring insoles) is employed in free-living conditions to measure the motor-related health status of the patients.

**For Parkinson’s disease**, there are many studies that use wearable devices similar in capabilities and body part-mounting position to the ALAMEDA PD study to quantify the level of tremor, freezing of gait or dyskinesias experienced by a patient. A wrist-worn accelerometer can be employed to develop a tremor stability index, which can, in turn, help distinguish between PD tremors and essential tremors with a good level of accuracy [49]. Parkinson’s tremor severity can also be quantified using wrist-worn devices, such as smartwatches [16,17], while accelerometers mounted below the ankle, on the sides of a shoe, can assess gait and analyze the turning capabilities for PD patients [50]. While the cited works employ a larger number of patients than the ALAMEDA PD study, they are all performed under laboratory conditions and for a limited duration in time.

Mobile health applications for PD that use a smartphone or a tablet enable the collection of patient-reported outcomes (motor fluctuations) and relevant biomarkers that can indicate disease severity [51]. The PD_Manager system [10], the mPower study [11] and the work by Zhan et al. [51] use smartphone applications to actively evaluate both motor and non-motor (such as anxiety/depression, dementia, orthostatic hypotension, and RBD) symptoms associated with Parkinson’s as measured by the scales of the MDS-UPDRS test. However, the ALAMEDA project is innovative since it provides the real everyday monitoring of the emotional status, exercise/dietary habits, social interaction and well-being of PD patients, providing a holistic approach. In the ALAMEDA PD study, two specialized tablet applications (the line-following and virtual supermarket tests) are employed every 3 months to comprehensively assess the cognition, including learning and memory, executive function and language capabilities of PD patients.

**In the stroke rehabilitation** case, there is significant interest in quantifying gait and upper limb movement parameters that can be indicative of improvements or regressions in patient motor deficits. A recent work reviewing the utilization of wearable technology to assess gait and mobility post-stroke [52] identified accelerometers, IMU-based activity monitors and pressure sensors as the most commonly used wearables to extract gait and mobility measures. The most often assessed metrics include gait speed and cadence for gait, and step count and duration of activity for mobility. However, as in the PD case, there are few research studies (e.g., [53]) that consider an analysis of outpatient or at-home registered ambulation data. Boukhennoufa et al. [54] observe only one study [15] out of 33 reviewed works, which computes its estimates under free living conditions (patients of a specialized rehabilitation care facility for which activities are recorded that are either part of therapy sessions or of normal living) and which averages about 9 days of recorded motion data per patient.

It is further the case that relatively few works (e.g., [55,56]) examine the validation and predictive power of accelerometer and IMU based sensors in use specifically for stroke patients (or patients with an impaired walking pattern in general). Peters et al. [52] note that gait abnormalities, such as inconsistent or slow stepping and walking speed and decreases in single limb stance, can limit the accuracy of some sensors (e.g., IMUs, specifically if worn at the hip or on the shin and ankle of the paretic leg). In contrast to this, the ALAMEDA stroke study employs easy-to-wear insoles, capturing the ground normal force component, in addition to IMU sensors worn on a belt and the wrist-worn accelerometer. Gait parameters extracted from the insoles (see Section 2.2.1) can improve actual step counts and stride times, as well as indicating imbalances between the left and right foot steps and thus better inform algorithms that seek to classify gait and balance issues. It is also the case that the novel combination of sensors used in ALAMEDA has the potential to validate the capability of accelerometer and IMU-based belts to accurately measure gait parameters in patients presenting with abnormalities by comparing to the higher-confidence measurements made by the insoles.

A long-term study comparable in objectives to the ALAMEDA stroke pilot is MOBITEC [12], which investigates aspects of quantitative gait analysis and balance assessment, lower-limb muscle power, general physical ability or life–space analysis (list and frequency of the most often visited locations apart from the home environment) of post-stroke patients at 3, 6, 9 and 12 months after the initial enrollment. However, data collection is limited to the clinical visits at the mentioned milestones, using standard medical tests and devices that can be used only in a lab setting (e.g., a force platform for balance testing). In contrast, the ALAMEDA stroke study aims to collect PRO data from patients, apart from objective information from wearables by means of a light schedule of questionnaires delivered through a chatbot interface.

**In the multiple sclerosis** case, a research program investigating the potential of wearable devices to help measure and predict clinical outcomes in neurological diseases (RADAR-CNS (https://www.radar-cns.org/, accessed on 11 August 2023)) highlighted the barriers and facilitators for mHealth technology that have been explored for MS [57]. Findings show that patients with a relapsing–remitting course of MS emphasize the importance of smartphone apps and wearable devices to collect symptoms patterns in between appointments in order to identify the occurrence of relapses. Moreover, multiple domains have emerged as potential targets to monitor through mHealth in MS (e.g., physical activity, diet, and physiological parameters). The ALAMEDA MS study envisions both aspects in its mission itself, as the main research question is related to the prediction of MS relapses through the collection of multiple-domain mHealth parameters.

Waist- and head-mounted IMU sensors can be used to determine unstable walking patterns and fatigue in MS patients [14]. Measures of variability and asymmetry in stride, step and pelvic sway are strongly correlated to pelvic compensations in walking, which in turn have a moderate correlation with MS severity as measured by the EDSS scale. The ALAMEDA MS study aims to quantify similar influences using a set of less-obstructive sensors, such as the belt-mounted IMUs and the insoles. The variability and asymmetry of gait measures can also be used to classify between moderate (EDSS score between 3 and 5) and advanced MS (EDSS score > 5), with the observation that the data were collected in a lab setting based on the 6 min walk test [58].

Recent efforts to collect long-term and diverse markers of MS severity (other than mobility-related ones) have highlighted the need to use PROs to obtain information that is important to patients who want to be better aware of their condition [13]. An example study resembling the ALAMEDA data collection protocol (though for a length of only one month) collects both PRO information and data from a Samsung Gear S2 smart watch [59]. Study findings show that objective metrics (e.g., max steps per day) can be moderately to strongly correlated to PROs, looking at experienced fatigue severity, the 2 min walk test, or patient-determined disease steps.

The ALAMEDA MS study clearly follows this line of reasoning by enabling the collection of PROs through a light schedule of standard medical questionnaires, as well as a set of relevant questions (see Table A2 in Appendix A) delivered through a chatbot interface.

From the perspective of the data collection journey itself, specifically in longer-term studies, researchers also looked at reasons causing patients to have low or non-existent engagement with digital PRO submission applications [60]. Health problems, technical barriers, feeling too busy or simply not wanting to submit data because of the lack of personal benefit are cited as the most frequently invoked reasons for dropping out of studies requiring PRO submission. Authors of [60] also note a strong correlation between the frequency of requested PRO submission and the dropout rate, specifically for longer studies, whereby a 90-day study with daily PRO collection has a three-times-higher dropout rate than a 12 month study performing only quarterly PRO collection.

The ALAMEDA studies data collection journey is well positioned to address these concerns. The inclusion and exclusion criteria of patient selection ensure that the health status itself cannot be a cause for missing PROs. The data collection journey of each pilot study provisions for an intense monitoring limited of 2 weeks in duration, implemented only at the 6- and 12-month marks for MS and stroke, and quarterly marks for PD. During the rest of the time, ALAMEDA pilots maintain a light schedule of PRO submission, enabling the postponing of responses and completion of questionnaires over several interaction sessions. Furthermore, the MS and stroke pilots make use of a chatbot interface to deliver most of the questionnaires and relevant questions, and studies indicate that patients could prefer the delivery of more complex or longer questionnaires through a chatbot interface [61]. It is also worth noting that the relevant questions were co-developed by clinicians and the main end users of the system, namely the patients and their caregivers. This process follows the guidelines developed within the MULTI-ACT project (https://www.multiact.eu/, accessed on 11 August 2023), which implemented a new model, allowing for the effective cooperation of all relevant stakeholders of health research.

These latter aspects position the ALAMEDA pilot study better from the perspective of not overburdening the daily life schedule of the patients. To reduce technical barriers and the burden of remembering how and when to wear the wearable devices and what needs to be reported, ALAMEDA implements a set of notification mechanisms, video tutorials explaining device usage (see Section 2.2.2) and checklist-style reminders to offer patients all the information required for sending relevant data either through PROs or the devices they use. Solutions similar to the above-mentioned ones, which increase compliance, improve usability and enable a higher quality of collected data, have also been highlighted as necessary features in a study looking at the usability of the mPower application for PD [62].

### 3.2. Use of Auxiliary Datasets

As described previously, the ALAMEDA pilot studies are in alignment with the objectives, practices and means for data collection used in the related literature. However, the ALAMEDA data collection journey also has a set of distinguishing characteristics. The pilot studies employ a novel combination of minimally intrusive wearable sensors, whose combined capabilities open up the prospect of a more informed analysis for the objectives set out in Section 2.4.1. Wrist-worn accelerometers cover the detection of physical rehabilitation exercises and intentional upper arm usage in stroke patients. Combined insole and belt-mounted IMUs cover gait and balance defect analysis. Furthermore, because for many of the wearable-based detection predictions there is a redundancy of information sources (e.g., gait parameters such as step count, cadence, stride asymmetry, measurable using insoles, wrist accelerometers and waist-mounted IMUs), the opportunity arises to validate the capability of one sensor in comparison to another, under free living conditions.

Furthermore, to overcome the challenge of a reduced number of enrolled patients per pilot, we make use of data collected in datasets where the used features have a substantial overlap with information collected in the ALAMEDA pilots. Table 11 lists a selection of prediction targets (that are also part of the ALAMEDA pilots—see Table A4, Table A5 and Table A6 in Appendix B), and the datasets they are extracted from, as well as the features and the machine learning models that will be employed for pre-training on the auxiliary dataset and transferring to data collected in the ALAMEDA pilots.

For PD, MDS-UPDRS Sections I and II, as well as PDQ-8 scores can be predicted based on two different setups. Using the mPower dataset, a longitudinal task is defined that uses demographics data, IMU data from walking tasks and PDQ-8 and MDS-UPDRS scores from previous evaluations. A modified version of a time series classification model (InceptionTime [63]) is used for the prediction. The alternative setup involves use of the FoxInsights dataset in a cross-sectional analysis, where the answers to survey questions concerning symptoms and daily activities (which are also partially covered in the ALAMEDA questions for PD) are used as features to a gradient-boosted tree regressor (CatBoost) model. Data from the Novel Loadsol insoles provide the same type of input (ground reaction force measurements) as those used in the gait in PD dataset, which is used to predict a Hoehn and Yahr score using an XGBoost regressor model.

For MS, the prediction of EDSS progression is also tackled from two perspectives. Using the MSOAC Placebo Dataset for pre-training, a CatBoost regressor predicts the score value at a future date using input from T25-FW, NHPT and previous EDSS evaluations as input (all of which are also collected in the ALAMEDA MS pilot). A simplified version of the problem entails a binary classification of the future EDSS score as stable or progressive by the use of the PROMOPROMS dataset. Many of the functional test questions in PROMPROMS have an equivalent in the relevant questions set for the MS pilot (see Table A2 in the Appendix A).

In the stroke study, the longitudinal early stroke cohort [67] is used as an auxiliary to pre-train MoCA score classification as normal (score ≥ 26), abnormal (20 ≤ score < 26) and dementia (score < 20). A fully connected layer network uses demographics input and window-based summaries of wrist-worn acceleremoter data to make the classification in a cross-sectional setup. Additionally, a longitudinal prediction setup is employed to perform an mRS score classification using demographics data and previous MoCA and mRS score evaluations on the Wearable-Based Walk Ratio Assessment in Healthy Adults and Chronic Stroke dataset [68].

### 3.3. Sleep Analysis

The ALAMEDA studies also place an emphasis on sleep monitoring for PMSS patients (and, in particular, for PD, where it is of major concern) to a higher degree than similar studies, whereby sleep-related data are collected using capable sensors (see sleep mattress described in Section 2.2.1) continuously during the project. To support and justify the sleep monitoring along the ALAMEDA, it is widely acknowledged that in the domain of neurodegenerative diseases, sleep disturbances are common, resulting mainly in fatigue, irritability, headaches, impaired motor and cognitive skills, depression, and daytime somnolence [69]. Sleep patterns are strongly interlinked with brain functionality and structure, intrinsically related to well-being and mental and physical health as highlighted in [70]. An apparent vicious circle of interchanging roles of poor sleep to increased risk of poor health takes place, limiting and simultaneously projecting sleep and health quality. It is well established that sleep disturbances/disorders are often among the first signs of distress [71], where common mental health problems, such us anxiety and depression [72], can often underpin sleep problems [73]. Nowadays, disorders of sleep are taken under serious consideration as part of neurodegenerative diseases, but for many years, they were not considered a relevant part [74]; specifically, sleep abnormalities are clearly recognized as a distinct clinical symptom of concern in neurodegenerative disorders [75]. More specifically, for the cases considered in ALAMEDA, in the PD case, sleep disorders are commonly encountered not only as consequences of the damage to the central nervous system but also from the applied treatment. The majority of PD patients suffer from excessive daytime sleepiness, and/or fragmented sleep [74,76,77]. As a result, up to 80% of PD patients report poor sleep, which has been characterized by shorter sleep periods and sleep efficiency [78]. In MS, patients report worsening of symptoms, such as daytime drowsiness, increased fatigue, decreased concentration and memory, and worsening depression due to lack of restful sleep [79,80]. Poor sleep is common in people with multiple sclerosis, with about 50% of people with MS reported to experience some form of sleep disturbance. In the case of stroke survivors, post-stroke sleep disorder (PSSD) [81,82] has a negative effect on the outcome and recovery from stroke, as poor sleep quality is associated with daytime sleepiness, reduced cognitive function, functional status, and even mortality [83,84]. PSSD is one of the frequently reported symptoms after a stroke, occurring in 21–77% of stroke patients [85].

As an auxiliary task for sleep analysis in ALAMEDA, sleep stage prediction is employed on data that have complete overlap with those collected using the Fitbit Smartwatch and the Withings sleeping mattress. Based on features such as activity level, heart rate, heart rate variance and SpO2 levels (on hand of the MESA dataset [86]), a recurrent network architecture is employed to classify the sleep stage of a 5 min window as REM, non-REM or awake.

An additional output of sleep analysis in ALAMEDA is based on unsupervised learning methods on data from the prototype ENORA mattress toppers. Using principal component analysis (PCA), a codebook of sleeping stances is created, which can be used to describe the mobility patterns of a person in their sleep. These mobility patterns can then be used as input to predictions for sleep quality metrics.

### 3.4. Non-Disease Related Data Analysis

A relevant element of novelty in the ALAMEDA pilot use cases is the study of the influence of non-disease-related factors (psychological, financial, societal or quality-of-life related) on the evolution of the patients’ health status. The underlying assumptions are that situations, such as financial difficulties, low education, unemployment due to the specific illness, absence of marital status or older age, are associated with a limited self-care capacity, increased mobility difficulties, a higher rate of depression and a considerable negative effect on the patients’ quality of life.

The ALAMEDA pilot studies aim to quantify the strength of these correlations and analyze the degree to which financial hardship and lack of social support are associated with worse QoL, independent of depression or anxiety levels. To do this, a 51-item socio-economic factors questionnaire is designed to collect information on demographic/residence data, socio-economic status, medical history and use of medication, as well as the use of health services. The questionnaires are administered to each enrolled patient by a medical professional, at the beginning of the study and at its milestone follow-up visits (i.e., at 6- and 12-month marks). At the end of the pilot studies, a correlation analysis is performed between the questionnaire responses and the results of the standard medical tests that capture general mobility and cognitive health dimensions (e.g., PDQ-39, Beck’s Anxiety and Depression Inventory, MOCA, MDS-UPDRS I—cognitive; EDSS, MDS-UPDRS III/IV, Romberg test and Berg balance scale—mobility).

An additional investigation looks at other non-disease-related factors which shape the quality of life of a patient and which may influence their ability to manage the disease. Specifically, ALAMEDA pilot studies will implement an automated system to collect statistical data on environmental factors (temperature, humidity, air pollution—particulate matter levels) from the local region of the patient residence. A questionnaire will be designed to gather information on patient habits (e.g., diet, smoking, active life—practicing a sport or a hobby), as well as their perceived levels of family support/inclusion. This questionnaire is to be administered by medical professionals at the half-way (6 month) and end marks of the studies. The overall aim is to analyze the predictive power of the environmental, habit and social relations data in predicting the patient health status, by including these variables in the input of algorithms that handle information presented in Section 2.4.2.

### 3.5. Pilot Study Challenges

In the design of the data collection journey for each pilot study considered in ALAMEDA, a set of challenges and mitigation actions are taken under account. The most important challenges in collecting a relevant set of data relate to the ability of patients to correctly use and interact with the wearable devices given to them, as well as complying with the schedule of PRO submissions enabled by the ALAMEDA Digital Companion.

In Table 12, we show that for each challenge, we provisioned for a technical mitigation solution as well as a process level one, which guides the experience of the patient using the devices and software applications.

The ALAMEDA study provisions for a usability and patient experience questionnaire whose questions follow the model of the unified theory of acceptance and use of technology (UTAUT) [87]. This questionnaire is administered at the mid and end-of-study milestones, asking in particular about the experience of interacting with the smartwatch and the ALAMEDA smartphone applications that are used in the continuous monitoring stream. Furthermore, clinicians receive weekly updates concerning the percentage of completed questionnaires that are due according to the schedule described in Table 7, Table 8 and Table 9.

At the time of writing, 14 PD patients, 6 MS patients and 6 stroke patients reached the first milestone for usability evaluation. Preliminary results showed that, for MS patients, the average monthly completion rate of PROs is close to 60% (with a minimum at 24% and a maximum of 92.5%). The enrolled stroke pilot patients are old and unfamiliar with the frequent use of technology, such that only two out of six patients are actively filling in PROs while not hospitalized or at a milestone evaluation. The stroke study team therefore intends to collect more frequent PRO responses during the intense monitoring periods, which happen under the supervision of medical personnel. PD patients are consistent in their monthly responses to PRO requests and show good adherence to the proposed collection protocol, completing a total number of more than 800 questionnaire instances.

In the initial responses to usability questionnaires, PD patients found the smart watch and the smartphone application unobtrusive and easy to use (with an average of 3.1 on a 0–4 Likert scale). However, patients signaled some difficulty in responding in the appropriate time to the daily questionnaires and considered the number of questions jarring. Meanwhile, MS patients considered the smartwatch useful in everyday life (average score of 4 on a 1–5 Likert scale) and easy to interact with. Patients expressed confidence in using the WellMojo questionnaire application (3.8 average on a 1–5 Likert scale) and found the interaction with the Conversational Agent to be sufficiently simple (an average of 2.7 on a 1–5 Likert scale asking for difficulty of interaction).

An important highlight to drive change in the ALAMEDA application functionality (especially for PD) is the desire of patients to make the feedback on their health status (as derived from the PRO and objective data they submit) more regular and easier to follow (expressed as a value of 2.7 on the 0–4 Likert scale). This is an avenue that we want to pursue with priority.

### 3.6. Privacy

The ALAMEDA pilot studies are observational in nature and rely on retrospective analysis to determine the predictive power of the collected data. As such, the collected datasets will only be made available to third-party entities at the end of the project. To ensure the privacy of users and their data, a detailed set of measures has been put into place.

All wearable devices are either CE marked or operate in a way that stores data locally on the device before it is retrieved and stored in a secure way in the ALAMEDA Research Data Management Platform. There is a detailed step-wise procedure for creating patient accounts, with use of the ALAMEDA Digital Companion that ensures that data are subsequently anonymized. All PRO data coming in from the component applications of the ALAMEDA Digital Companion are stored on servers owned by members of the ALAMEDA consortium, for which a secure data management plan is in place.

Upon entering the project exploitation phase, the exact method of obtaining access to the collected data will be made available. Each pilot study received the Ethics Committee approval of each organization, and each study participant signed their informed consent upon enrollment.

### 3.7. Impact of Digital Transformation on Patient Health Monitoring

Considering the negative burden of the pathologies included in ALAMEDA, digital devices can help with correct monitoring of the patient over time, predicting worsening, avoiding relapses and anticipating the effect of treatments. Importantly, the value-based healthcare model was adopted to better treat patients affected by neuro-degenerative disorders and to reduce costs. In fact, the goal of the model is to enable the healthcare system to create more value for patients and to increase their quality of life.

To try to address this need, a new vision must be adopted, and the value of the scientific outcomes must be assessed by different dimensions. This was reached by the EU-funded MULTI-ACT project that carried out the Collective Research Impact Framework (CRIF) [88]. The result was the development of five dimensions useful for evaluating several pathologies, including PD, MS and stroke. Therefore, the MULTI-ACT methodology represents a general framework to be used in the ALAMEDA project to assess the impact of research and innovation in three different pilots, where the innovation part is represented by the digital devices, and the aim is to assess their impact on patients with PD, MS and stroke. In the presented pilot study setups, we exploited two of these five dimensions (efficiency and social dimension) to set up KPIs specific to the three diseases considered and the three pilots (Greece, Italy and Romania), where the studies are conducted by using digital devices. More specifically, several KPIs for the efficiency part were set up in order to monitor the patients with devices over time and to evaluate the related costs (e.g., days of nurse per patient, and days of work loss). Other KPIs for the social part were developed in order to provide evidence about the impact of tools used in ALAMEDA on the lives of patients and their families (e.g., days of work lost by caregivers). Thus, remote patient monitoring clearly emerges as a favorable strategy, which is strongly underlined by the European Innovation Council as one of the breakthrough health innovation strands to improve healthcare technologies and the quality of life of patients.

### 3.8. Study Limitations

The main limitation of the ALAMEDA study is the low number of participants per pilot study, which prevents performing the ML-based prediction solely based on data collected in ALAMEDA, requiring instead to make use of model adaptation or pretraining as explained in Section 3.2. However, as the main objective of the study is to validate the prediction potential for the novel data stream combinations introduced in ALAMEDA, future studies can expand on the number of participants and focus on the prediction setups, which we identify as having the largest explanatory capability.

In the PD pilot study, we acknowledge that the use of different wearable devices may impact the variability of the data, and the limited number of converters may affect the generalizability of the results. The PD study starts as a single-point observational study and allows patients willing to participate in longitudinal observations at six, nine, and twelve months from the initial observation. Although data from the wearable sensors are recorded for these additional visits, the meaningful longitudinal analysis could potentially not be conducted because the participants can have several changes in medications and dosage over the course of the study, and the number of PD patients is too small to attempt the analyses of smaller groups in which these parameters are constant over the longitudinal duration. Larger and longer-duration studies are required to replicate these findings and to evaluate how they change over time.

In the MS pilot study, a limitation is related to the long period of monitoring the participants. Over 12 months, several daily life and disease-related drawbacks may occur, limiting adherence or leading to drop out. However, some clinical questions, such as those addressed by ALAMEDA, are often better addressed using real-world data integrating disease history, performance tests, patient-reported outcome measures and data from wearable devices [89]. Here, we adopted different mitigation procedures to minimize missing data and drop-out rates (e.g., engagement of patients in peer-support groups following MULTI-ACT guidelines), but data standardization and validation within datasets, harmonization across datasets, and the application of appropriate analysis methods are important considerations to take into account for present and future studies [89].

From the perspective of the stroke pilot study, the short period of follow-up regarding the cognitive impairment constitutes a limitation of the study. A further concern is represented by the impossibility of including stroke patients with severe neurological deficits, as they are unable to use the ALAMEDA system.

## 4. Conclusions

This paper introduces the protocol of data collection and analysis of predictive capability for the PD, MS and stroke studies taking place within the ALAMEDA Project. The set of wearable devices and software applications which enable the retrieval of objective and patient-reported information were outlined together with a clear schedule for the collection of the data. Patients enrolled in the pilot studies undergo 1–2-week-long intense monitoring (using all devices) at milestone periods, while also submitting PROs for the entire 1-year duration of the studies.

Clear prediction targets are identified for data from wearable devices (specifically with respect to movement and sleep), while novel, ML-based prediction setups are proposed for exploration, taking advantage of the input from both wearable data summaries, as well as patient reported outcomes. The prediction setups include both longitudinal as well as cross-sectional analyses.

There are three main innovation contributions brought by the ALAMEDA studies. First, a novel combination of wearable devices (wrist-worn accelerometer, IMU-based belt sensors and smart insoles, and under-mattress sleep sensor) will be used to analyze mobility metrics, as well as motor and sleep disorders according to the needs of each pilot study. The wear locations are typical (wrist, waist and hips, and in the shoes) for the bracelet, belt and insoles, while the sleep sensor needs only to be placed underneath the usual sleeping mattress. These locations align with indications from the literature and contribute a minimal overhead for the patients. While the size of the ALAMEDA patient cohorts is relatively small compared to related studies, we compensate for this through the exploitation of the repeated intense monitoring periods, which are set 3 months apart and which enable the focus on analyzing the change in metrics and predictions over time. For certain analysis tasks (e.g., gait and sleep metric extraction), the selected sensors provide redundant detection capabilities such that the studies enable a comparative performance analysis of sensing modalities.

Second, the ALAMEDA pilot studies will collect PRO data for the duration of a year using smartphone applications that exploit a traditional questionnaire interface, as well as a modern Conversational Agent interface. The flexibility in interfaces is intended to prolong patient engagement for the duration of the studies. Apart from motor difficulties, the PROs capture information about the cognitive-, emotional- and quality-of-life-related aspects of the patient status. The proposed analysis for PRO data exploits the duration of the collection period by looking at changes in the correlation to standard medical tests performed at the milestone clinic visits.

Third, the ALAMEDA studies will supplement collected health status information with non-disease-related factor analysis, aiming to analyze the predictive power of quality-of-life, economic status- and environment-related data to explain observed changes in the patient health status.

Altogether, the ALAMEDA studies will lead to a better understanding of the dynamics and predictability of changes in the health condition of a PMSS patient, as measured by the current standard medical tests in the analysis of PD, MS and stroke. Multiple objective and patient-reported sources will be used to quantify the changes, and the studies will reveal the predictive power of each source in particular, as well as their novel combination, thereby informing future efforts to improve long-term treatment options for PMSS patients.

## Figures and Tables

**Table 1:** Inclusion and exclusion criteria for the ALAMEDA PD pilot study.

Inclusion Criteria	Exclusion Criteria
- Diagnosis of PD [26] - Age 30–75 - Advanced PD, as defined by the presence of even minor motor complications (fluctuations or dyskinesias) - H&Y 2.5 or less at “on” phase - cooperative-excited about participating in study-motivated - ability to use smart devices - Cognitively intact (MOCA Score ≥ 25) - able/has the means to return for re-evaluation to our clinic at 3-monthly intervals	- Psychiatric comorbidity (psychosis, major depression) that may interfere with his/her ability to engage in the study - Significant comorbidities (orthopedic, cardiovascular, respiratory, etc.) that may restrict ADLs. - Not able to follow instructions regarding the application and use of sensors, including the necessary interactive components - Presence of Dementia

**Table 2:** Inclusion and exclusion criteria for the ALAMEDA MS pilot study.

Inclusion Criteria	Exclusion Criteria
- Age 18–45, males and females - Definite diagnosis of relapsing-remitting MS according to the revised 2017 McDonald criteria; - Score of less than or equal to 4 on the Expanded Disability status Scale (EDSS) - Being relapse free in the last month - Acquaintance to smartphones and technology use availability of reliable internet connection - Signed consent form	- Psychiatric comorbidity (psychosis, majordepression) that may interfere with the ability to engage in the study -Significant neurological or orthopedic comorbidities - Not able to follow instructions regarding the application and use of sensors, including the necessary interactive components - Severe cognitive deficit (MOCA Score < 25)

**Table 3:** Inclusion and exclusion criteria for the ALAMEDA stroke pilot study.

Inclusion Criteria	Exclusion Criteria
- Age 18–85 years - Hospitalized for stroke in the last month - Ability to use smart devices - Patient is able/has the means to return for reevaluation and to be closely monitored during at-home neuro-rehabilitation	- Aphasia - Complete bilateral blindness - Patients who have plegic limbs with 0/5 points on the MRC scale or severely impaired muscle strength of less than 3/5 points on the MRC scale - Patients that are completely non-ambulatory at the time of their hospital discharge - Patients with severe neurocognitive disorders that score less than 10 points on a MOCA questionnaire taken before discharge from the hospital

**Table 4:** List of ALAMEDA devices and their intended use.

Device	Model	Usage Method (Continuous vs. Limited Duration)	Extracted Metrics
Smart Watch	Fitbit Versa Lite Fitbit Charge 4	continuous	activity: no. of steps, intensity level periods, distance traveled, burned calories sleep: sleep stage durations (light, deep, and REM), sleep efficiency general: heart rate, blood oxygen levels
Smart Bracelet	ActivInsights GENEActiv	limited duration	activity summary: no. of steps, intensity level periods sleep: basic sleep stages, sleep efficiency raw accelerometer data: gait/balance issue classification
Smart Insoles	Novel Loadsol-ap	limited duration	gait metrics: no. of steps, cadence, step cycle time, loading rate, factor of imbalance raw plantar force data: gait or balance issue classification
Smart Belt	NTNU Prototype	limited duration	raw IMU data: basic activity recognition, gait/balance issue classification
Under Mattress Sleep Sensor	Withings Sleep Mat	limited duration (A limited supply of sleep mattresses is available for the MS and Stroke pilot studies. An extended but still limited duration use will be facilitated for these cases)	sleep stage durations, sleep efficiency, sleep apnea and snoring detection
Mattress Topper	ENORA Prototype	limited duration	sleep position heatmap, environment temperature, light level, sound level

**Table 5:** Prediction objectives for raw data collected from wearable devices.

Prediction Target	Pilot Study	Description	Input Devices
Tremor, Dyskinesia and Hypokinesia detection	PD	Real-time detection of tremor, hypokinesia and dyskinesia episodes based on unlabelled data under free-living conditions - onset and the end of each episode - episode duration - number of each type of episodes per day	NTNU Smart Belt GENEActiv bracelet - attached to wrist of most affected arm, ankle or shank Fitbit smartwatch
Restless Leg Syndrome Detection	PD, MS, stroke	Real-time detection of “restless leg” episodes during sleep	GENEActiv bracelet - attached to ankle or shank NTNU IMU sensor - attached to ankle or shank
Physical Rehabilitation Exercise Detection	Stroke	Real-time detection and classification of rehabilitation exercise execution	GENEActiv bracelet - attached to wrist NTNU Smart Belt

**Table 6:** Monitoring protocols for ALAMEDA pilot studies.

Data Collection Protocol	Description	Time Period	Used Devices
Continuous Monitoring	Data collection process happening continuously throughout the study, involving: - precisely scheduled PROs - activity monitoring using Smart Watch - sleep monitoring using Smart Watch	Throughout pilot study	Smartphone for PROs Fitbit Smart Watch
Intense Monitoring Period	Data collection process that happens over 1–2 weeks prior to a study milestone, involving the use of all available devices and requests for specific patient activities.	- 1 week prior to every 3-month milestone (PD) - 2 weeks prior to every 6-month milestone (MS) - 2 weeks prior to every 6-month milestone (Stroke)	Smartphone for PROs Fitbit Smart Watch Withings Sleep Mat GENEActiv bracelet NTNU Smart Belt Novel Loadsol Insoles ENORA Mattress Topper

**Table 7:** PD pilot data collection journey—list of monitored variables and their method of collection.

Variable	Description	Data Value Range	Acquisition Method	Used Devices
**Domain I—Mobility, general motor or physical function**				
Step count, periods of relative immobility/slowness of movement	Continuously monitored step count and other features of general mobility in daily life	step count: integer (0–15,000) periods of mobility: seconds (0–14,400)	Continuous Monitoring - eHealth device	Fitbit Smart Watch
Heart rate, SpO${}_{2}$ levels	Monitoring of daily heart-rate and blood-oxygen levels	heart rate: integer (30–200) blood-oxygen levels: percentage (0–100)	Continuous Monitoring - eHealth device	Fitbit Smart Watch
Physical Activity Amount	Exact time periods of inactive, light, medium or vigorous activity	seconds (0–14,400)	Intense Monitoring every 3 months - eHealth devices	Fitbit Smart Watch GENEActiv Bracelet
30 min intense walk	Intense, outdoor half-hour walk using smart watch, bracelet, belt and insoles	all metrics from previous rows avg. cadence per 30 s: integer loading rate: N/s (speed of normal force applied to body) factor of imbalance: percentage (disproportion of load between feet) peak force: N (maximum force push while walking)	Intense Monitoring every 3 months - eHealth devices	Fitbit Smart Watch GENEActiv Bracelet NTNU Smart Belt Loadsol Insoles
OAB-Q	Assess subjective perception of bladder problems	integer, questionnaire score: 6–48	Continuous Monitoring - PRO on smartphone (every month)	Smartphone
MDS-UPDRS II	Scale of report of ADLs based on motor activities	integer, questionnaire score	Continuous Monitoring - PRO on smartphone (every week)	Smartphone
MDS-UPDRS IV	Levels of motor fluctuations and dyskenisias	integer, questionnaire score	Continuous Monitoring - PRO on smartphone (every week)	Smartphone
**Domain II—Sleep disorders**				
Pittsburgh Sleep Quality Index (PSQI)	Self-administered questionnaire to assess sleep patterns	integer, questionnaire score: 0–21	Continuous Monitoring - PRO on smartphone	Smartphone
General Sleep Patterns	Continuous monitoring of general sleep stage duraitons using smart watch and under mattress sensor	total bed time: hours (0–12) light sleep: hours (0–12) deep sleep: hours (0–12) REM sleep: minutes (0–240) apnea: boolean (true/false) snoring: minutes (0–240)	Continuous Monitoring - eHealth devices	Fitbit Smart Watch Withings Sleep Mat
Intense Sleep Monitoring	Sleep monitoring during pilot milestones using eHealth devices and a polysomnograph	Previous row metrics + polysomnography analysis	Intense Monitoring every 3 months - eHealth devices	ENORA Sleep Mat Fitbit Smart Watch GENEActiv Bracelet
**Domain III—Mental and cognitive ability**				
PDQ	Self-report measure of cognitive dysfunction, investigating: attention, retrospective memory, prospective memory, and planning	integer, questionnaire score: 0–80	Continuous Monitoring - PRO on smartphone (every month)	Smartphone
Keystroke dynamics	Detailed timing of typing on smartphone	Enum: classes of abnormal typing patterns	Continuous Monitoring - eHealth devices	Smartphone
Line Tracking Test	Self-administered test on tablet to assess various aspects of arm/hand movement	Reaction time: ms Movement time: msec Internal time delays: msec	Intense Monitoring every 3 months - eHealth devices	Tablet
Virtual Supermarket Test	Self-administered test based on a 3D serious game to assess cognitive decline	time to completion: ms (scores above 215,000 ms indicate possible cognitive impairment)	Intense Monitoring every 3 months - eHealth devices	Tablet
**Domain IV—Emotional status**				
Facial Expression Analysis	Estimate mood using facial expression analysis enabled by MEAA (see Section 2.2.2)	Enum: mood class and probability	Continuous Monitoring - eHealth device	Smartphone
PHQ-9	Monitor the severity of depression and response to the treatment	integer: questionnaire score (0–27)	Continuous Monitoring - PRO on smartphone (every month)	Smartphone
**Domain V—Quality of life and daily living**				
MFIS	Assessment of the effects of fatigue in terms of physical, cognitive and psychosocial functioning	integer: questionnaire score (0–84)	Continuous Monitoring - PRO on smartphone (every month)	Smartphone
Food Habits Questionnaire (FH-Q)	Self-report questionnaire measuring food intake habits about typical eating patterns over the past month	integer: questionnaire score (0–18)	Continuous Monitoring - PRO on smartphone (every month)	Smartphone
MDS-UPDRS I	Partial (patient-reported) assessment of non-motor aspects of experiences of daily living	integer: questionnaire score (0–24)	Continuous Monitoring - PRO on smartphone (every week)	Smartphone

**Table 8:** MS pilot data collection journey—list of monitored variables and their method of collection.

Variable	Description	Data Value Range	Acquisition Method	Used Devices
**Domain I—Mobility, general motor or physical function**				
Step count, periods of relative immobility/slowness of movement	Continuously monitored step count and other features of general mobility in daily life	step count: integer (0–15,000) periods of mobility: seconds (0–14,400)	Continuous Monitoring - eHealth device	Fitbit Smart Watch
Heart rate, SpO${}_{2}$ levels	Monitoring of daily heart rate and blood oxygen levels	heart rate: integer (30–200) blood-oxygen levels: percentage (0–100)	Continuous Monitoring - eHealth device	Fitbit Smart Watch
Physical Activity Amount	Exact time periods of inactive, light, medium or vigorous activity	seconds (0–14,400)	Intense Monitoring every 6 months - eHealth devices	Fitbit Smart Watch GENEActiv Bracelet
6 min walk test	Sub-maximal exercise test assessing walking endurance and aerobic capacity. Participants walk around an indoor perimeter for a total of six minutes.	metrics from first row avg. cadence per 30 s: integer loading rate: N/s (speed of normal force applied to body) factor of imbalance: percentage (disproportion of load between feet) peak force: N (maximum force push while walking)	Intense Monitoring every 6 months - eHealth devices	Fitbit Smart Watch Loadsol Insoles
MSWS-12	12-item self-report measure on the impact of MS on walking ability	integer, questionnaire score: 12–60	Continuous Monitoring - PRO on smartphone (every 2 weeks)	Smartphone
OAB-Q	Assess subjective perception of bladder problems	integer, questionnaire score: 6–48	Continuous Monitoring - PRO on smartphone (every month)	Smartphone
AMSQ	Unidimensional 31-item questionnaire for measuring of arm function in MS	integer, questionnaire score: 31–186	Continuous Monitoring - PRO on smartphone (every 2 weeks)	Smartphone
**Domain II—Sleep disorders**				
Pittsburgh Sleep Quality Index (PSQI)	Self-administered questionnaire to assess sleep patterns	integer, questionnaire score: 0–21	Continuous Monitoring - PRO on smartphone (every month)	Smartphone
General Sleep Patterns	Continuous monitoring of general sleep stage durations using the smart watch	total bed time: hours (0–12) light sleep: hours (0–12) deep sleep: hours (0–12) REM sleep: minutes (0–240) apnea: boolean (true/false) snoring: minutes (0–240)	Continuous Monitoring - eHealth devices	Fitbit Smart Watch
Intense Sleep Monitoring	Sleep monitoring during pilot milestones using eHealth devices and a polysomnograph	Previous row metrics + polysomnography analysis	Intense Monitoring every 6 months - eHealth devices	ENORA Sleep Mat Withings Sleep Mat Fitbit Smart Watch GENEActiv Bracelet
**Domain III—Mental and cognitive ability**				
PDQ	Self-report measure of cognitive dysfunction, investigating: attention, retrospective memory, prospective memory, and planning	integer, questionnaire score: 0–80	Continuous Monitoring - PRO on smartphone (every month)	Smartphone
Keystroke dynamics	Detailed timing of typing on smartphone	Enum: classes of abnormal typing patterns	Continuous Monitoring - eHealth devices	Smartphone
Line Tracking Test	Self-administered test on tablet to assess various aspects of arm/hand movement	Reaction time: ms Movement time: msec Internal time delays: msec	Intense Monitoring every 6 months - eHealth devices	Tablet
Virtual Supermarket Test	Self-administered test based on a 3D serious game to assess cognitive decline	time to completion: ms (scores above 215,000 ms indicate possible cognitive impairment)	Intense Monitoring every 6 months - eHealth devices	Tablet
**Domain IV—Emotional status**				
Facial Expression Analysis	Estimate mood using facial expression analysis enabled by MEAA	Enum: mood class and probability	Continuous Monitoring - eHealth device	Smartphone
PHQ-9	Monitor the severity of depression and response to the treatment	integer: questionnaire score (0–27)	Continuous Monitoring - PRO on smartphone (every month)	Smartphone
**Domain V—Quality of life and daily living**				
MFIS	Assessment of the effects of fatigue in terms of physical, cognitive and psycho-social functioning	integer: questionnaire score (0–84)	Continuous Monitoring - PRO on smartphone (every month)	Smartphone
Food Habits Questionnaire (FH-Q)	Self-report questionnaire measuring food intake habits about typical eating patterns over the past month	integer: questionnaire score (0–18)	Continuous Monitoring - PRO on smartphone (every month)	Smartphone

**Table 10:** Conditions for triggering alerts on subset of variables from the data collection journey.

Variable	Description	Pilot Study	Alert Conditions
**Domain I—Mobility, general motor or physical function**			
Step count, periods of relative immobility/slowness of movement	Continuously monitored step count and other features of general mobility in daily life	PD, MS, Stroke	Reduction of week average ≥ 20%
Self-assessed questionnaire for muscle tone	Self-assessed questionnaire to quantify the muscle tone variable	Stroke	Increase of ≥1 point compared to previous month
ACTIVLIM questionnaire	Self-assessed questionnaire to examine both upper and lower limb muscle strength using daily living activities	Stroke	When answer to a question changes from “easy” to “difficult” or “impossible”, compared to previous month
Dizziness and Balance questionnaire	Self-assessed questionnaire for the balance variable	Stroke	When a patient checks a symptom that was not checked in the previous month
**Domain II—Sleep disorders**			
Pittsburgh Sleep Quality Index (PSQI)	Self-administered questionnaire to assess sleep patterns	PD, MS, Stroke	Change in answer to item 5, marking an increase in occurrence frequency compared to previous month
**Domain III—Mental and cognitive ability**			
Virtual Supermarket Test	Self-administered test based on a 3D serious game to assess cognitive decline	PD	Deterioration of performance by ≥20% compared to test 3 months ago
**Domain IV—Emotional status**			
PHQ-9	Monitor the severity of depression and response to the treatment	MS, Stroke	Score increase of ≥1 point compared to previous month
**Domain V—Quality of life and daily living**			
MFIS	Assessment of the effects of fatigue in terms of physical, cognitive and psychosocial functioning	PD, MS, Stroke	Score increase of ≥16 points or ≥19% compared to previous month

**Table 11:** Prediction targets and the use of auxiliary datasets to pre-train ML models.

Disease	Type of Prediction	Support Dataset	Used Features	ML Model
PD	MDS-UPDRS I/II score prediction	mPower [11]	Demographics, Walking Task, PDQ-8 and MDS-UPDRS Surveys	InceptionTime [63] time series convolution model
PD	PDQ-8 score prediction	mPower		
PD	MDS-UPDRS II score prediction	FoxInsights [64]	Answers to survey questions	CatBoost Regressor (https://catboost.ai/en/docs/, accessed on 11 August 2023)
PD	PDQ-8 score prediction			
PD	Hoehn and Yahr score	Gait In Parkinson’s Disease [65]	Vertical Ground Reaction Force	XGBoost
MS	EDSS progression	MSOAC Placebo Dataset [66]	T25-FW, NHPT, EDSS from previous evaluations	CatBoost Regressor
MS	EDSS progression classification (stable vs. progressive)	PROMOPROMS Dataset [13]	demographics, Functional Test Questions (ABILHAND, Edinborough Inventory, Functional Independence Measure), Emotational Status Questions (Hospital Anxiety and Depression Scale), QoL questions (Life Satisfaction Index), MFIS	CatBoost Classifier
Stroke	MoCA score classification	Longitudinal Early Stroke Cohort [67]	Demographics and wrist-worn accelerometer data from daily life	DNN with fully connected layers
Stroke	mRS score classification	Wearable-Based Walk Ratio Assessment in Healthy Adults and Chronic Stroke [68]	Demographics, MoCA and mRS at previous evaluations	DNN with fully connected layers

**Table 12:** ALAMEDA data collection journey challenges and mitigation actions.

Challenge	Technical Mitigation	Process Level Mitigation
Patients find it challenging to fill in PROs	The ALAMEDA Digital Companion issues notifications and instructions on how to fill in each questionnaire	Medical professionals perform a dry-run of receiving a notification and completing a PRO while the patient is hospitalized/on the first days after enrollment
Patients find it challenging to follow the schedule for PRO submission	Notifications in the ALAMEDA Digital Companion can be postponed; Questionnaires can be split into multiple completion sessions	A light PRO schedule is provisioned: no more than 1 questionnaire a week or 1–2 relevant questions a day
Patients find it challenging to wear and operate many devices (e.g., smart insoles, smart belt) during the intensive monitoring period	An early morning checklist (comprising video tutorials of how to wear and use the devices) is delivered through the ALAMEDA Digital Companion every day of the intense monitoring period	Collect usability feedback at each pilot milestone and iterate over support documentation; Enable option for admission into clinic during the intense monitoring period (e.g., for stroke patients)
Patients find it challenging to understand their role and contribution in the study leading to reduced engagement and possible dropout	Patients can see their own data in the ALAMEDA Digital Companion; Social Media groups can be created to facilitate inter-patient support	Medical professionals keep a close contact with the patient; Medical professionals explain the pioneering role that patients play in the study: for themselves, as well as for future patients

## Data Availability

No new data were created or analyzed in this study. Data sharing is not applicable to this article.

## Appendix A. Set of Relevant Questions for Each of the ALAMEDA Pilot Studies

This section provides details on the set of relevant questions and their scheduling (frequency of expected answers), which constitute complementary patient reported outcomes (PROs), apart from the answers to standard questionnaires.

**Table A1:** List of relevant questions by health domain for the PD study.

Question	Intent	Possible Answers	Scheduling
**Domain I—Mobility, general motor or physical function**			
During the day today, regarding “off” periods, you have had:	Intensity and impact of experience of off periods	0, 1, 2, 3, or 4 0: None 1: very minor 2: Mild 3: Modest 4: Severe	Continuous Monitoring: End of each day
During the day today, regarding dyskinesias, you have had:	Intensity and impact of experience of dyskinesias	0, 1, 2, 3, or 4 0: None 1: very minor 2: Mild 3: Modest 4: Severe	Continuous Monitoring: End of each day
During the day today, regarding “off” periods, you have had:	Intensity and impact of experience of off periods	0, 1, 2, 3, or 4 0: None 1: very minor 2: Mild 3: Modest 4: Severe	Continuous Monitoring: End of each day
During the day today, regarding dyskinesias, you have had:	Intensity and impact of experience of dyskinesias	0, 1, 2, 3, or 4 0: None 1: very minor 2: Mild 3: Modest 4: Severe	Continuous Monitoring: End of each day
PD calendar: What description (A, B, C, or D) best describes your condition over the last half hour?	Length of periods of motor complications	A (bad motor condition), B (mediocre motor condition), C (good motor condition), D (bothersome dyskinesias)	Intense Monitoring (1 week): Every 30 min, during waking hours
**Domain IV—Emotional status**			
Which face/answer best describes your emotional state today?	Gestalt feeling of the day	Happy, Sad, Normal, Content, Disappointed, Frustrated	Continuous Monitoring: End of each day
How would you rate these statements over the past week? - I feel irritable - I feel lonely or isolated - I feel calm - I feel that I am full of energy - I feel safe and protected - I am under pressure from other people - I enjoy myself - I feel terrified or afraid - I feel discouraged about the future - I feel worried about my physical condition	Rating of feelings about things over a period of one week	Scale of 0–4	Continuous Monitoring: End of the week
**Domain V—Quality of life and daily living**			
How would you rate these statements over the past week? - I spent pleasant time with friends. - I shared my status with friends through messages, social media or phone calls. - I spoke with persons in the neighborhood and got information on what is going on. - I spent pleasant and relaxing time with family through meals, chatting, etc. - I went to the gym or had a dedicated time for exercise at home or outdoors - I read for pleasure or knowledge - I performed enjoyable outdoor activities - I went out for shopping or other chores - I performed chores at home	Pattern of social interactions linked to Quality-of-Life	Scale of 0–4	Continuous Monitoring: End of the week

**Table A2:** List of relevant questions by health domain for the MS study.

No.	Question	Intent	Possible Answers	Scheduling
	**Domain I—Mobility, general motor or physical function**			
1	In the last few days, have you noticed a sudden lack of strength in one or more limbs?	Intensity of upper and lower limb mobility issues	Yes/No	Continuous Monitoring: Once a week
1a	In which situation (e.g., carrying shopping, bags, or folders, picking up children or walking)?	If 1 = Yes	Free text	
1b	Did these disorders occur in limbs that did not previously have deficits?		Yes/No	
1c	How long did they last?		- A few hours or less - About a day - Two to four days - Most of the week	
2	Have you felt more rigid or less fluid in your movements in the last few days?	Intensity of rigid movement events	Yes/No	
2a	In which situation (e.g., carrying shopping bags, picking up children, walking)?	If 2 = Yes	Free text	
2b	Did these disorders occur in limbs that did not previously have deficits?		Yes/No	
2c	How long did they last?		- A few hours or less - About a day - Two to four days - Most of the week	
3	In the last few days, how have you felt when you move?	Frequency of near-fall events and balance issues	- Sure step - I have to pay more attention or stop to do other things-Unstable - Real risk of falling	
4	How many times have you stumbled over the last few days?		Numeric entry	
5	How many times have you fallen in the last few days?		Numeric entry	
6	In the last few days, have you noticed an alteration in sensitivity (e.g., arms or legs asleep, tingling, burning, unusual sensation to the touch)?	Intensity and frequency of upper or lower limb incidents	Yes/No	
	**Domain II—Sleep**			
1	Do you usually have trouble falling asleep (more than 30 min)?	Determine sleep quality	Yes/No	Continuous Monitoring: Once a month
2	Would you define your sleep “restful”?		Yes/No	Continuous Monitoring: Once a month
3	Have you ever been told, or suspected yourself, that you seem to “act out your dreams” while asleep (e.g., punching, arm flailing, making running movements, etc.)?		Yes/No	Continuous Monitoring: Once every 3 months
	**Domain III—Mental and Cognitive Ability**			
1	In the last few days, did you go through the information you need over and over again in order to remember it?	Quantify short-term memory and focus capabilities	- Never - Rarely - Sometimes - Often	Continuous Monitoring: Once a week
2	From 1 to 10 how hard are you struggling to stay focused on what you are doing (e.g., losing your train of thought, listening to what others are saying, reading a book or watching a movie)?	Quantify memory and focus capabilities	Scale 1–10	Continuous Monitoring: Once a week
2a	In which situation does it happen most frequently?	If answer to Q2 ≥ 6	Free text	
3	When an answer needs to be given, do you need to take an extra moment to pick up the thread and provide the best answer?	Quantify memory and focus capabilities	- Never - Rarely - Sometimes - Often	Continuous Monitoring: Once a week
4	During the day, do you have the feeling of having a full head that leads you to be less lucid?	Quantify issues in reasoning and task completion	- Never - Rarely - Sometimes - Often	Continuous Monitoring: Once a week
5	From 1 to 10 how difficult is it to find the word in your head? How often do you get the terms wrong?		Scale 1–10	Continuous Monitoring: Once a week
5a	In which situation does it happen most frequently?	If answer to Q5 ≥ 6	Free text	Continuous Monitoring: Once a week
6	How many appointments or commitments have you forgotten in the last two weeks?	Quantify long-term memory	Numeric entry	Continuous Monitoring: Once every 2 weeks
7	In the last two weeks, did you need to write down commitments more than usual to remember them (e.g., using extra notes or alarms)?		Yes/No	Continuous Monitoring: Once every 2 weeks
	**Domain IV—Emotional status**			
1	How are you feeling today?	Feeling of the day	One of: Anger, Fear, Sadness, Joy, Contempt, Cheer, Shame, Anxiety, Disappointment, Irritation, Serenity, Gratitude, Grudge, Resignation, Hope, Nostalgia	Continuous Monitoring: Once a week (also able to submit at will using chatbot interface)
2	Are you feeling stressed this week?	Intensity and frequency of stress	Yes/No	
2a	In which statement do you recognize yourself most?	If answer to Q2 == Yes	- Level 1: “daily life” stress (work, home, family) - Level 2: stress from overlapping commitments and difficulties in managing things to do with respect to my mental energies - Level 3: stress from the onset of worries or aggravation of existing ones (e.g., economic difficulties, conflicts at work or in the family) - Level 4: stress from strong destabilizing events (e.g., a radical change of life, bereavement)	
3	In the last week, have you experienced symptoms of previous relapses again?	Quantify relapse risk	Yes/No	
	**Domain V—Quality of Life and daily living**			
1	In the last month, have you had to give up social activities (eg. going out with friends, dinners with relatives, attending events, etc.) due to MS?	Pattern of social interactions linked to Quality-of-Life	- Never - Rarely - Sometimes - Often	Continuous Monitoring: Once a month
2	In the last month, have you had a greater need forassistance in carrying out your daily activities?			

**Table A3:** List Table of relevant questions by health domain for the stroke study.

Question	Intent	Possible Answers	Scheduling
**Domain III—Mental and Cognitive Ability**			
In the last few days, did you find it difficult toconcentrate on a task?	Intensity and impact of moments with loss of focus	- Never - Rarely - Sometimes - Often	Continuous Monitoring: Once a month
**Domain IV—Emotional status**			
How are you feeling today?	Feeling of the day	One out of: - Anger - Fear - Sadness - Joy - Contempt - Cheer - Shame - Anxiety - Disappointment - Irritation - Serenity - Gratitude - Grudge - Resignation - Hope - Nostalgia	Continuous Monitoring: Once a month (can also be provided at will, using chatbot interface)
In the last 4 weeks have you felt nervous, anxious, or on edge?	Intensity of negative feelings or worrisome attitude	- Never - Rarely - Sometimes - Often	Continuous Monitoring: Once a month
In the last 4 weeks have you worried too much about different things related to the stroke you suffered?			
**Domain V—Quality of life and daily living**			
In the last month, have you had to give up social activities (e.g., going out with friends, dinners with relatives, attending events, etc.) due to STROKE?	Pattern of social interactions linked to Quality-of-Life	- Never - Rarely - Sometimes - Often	Continuous Monitoring: Once a month
In the last month, have you had a greater need for assistance in carrying out your daily activities?			

## Appendix B. Prediction Targets and Ground Truth Conditions

**Table A4:** Prediction targets and ground-truth conditions for medical tests in motor ability domain.

Variable	Description	Pilot Study	Prediction Target and Condition
**Domain I—Mobility, general motor or physical function**			
6 min walk test	Exercise test assessing walking endurance and aerobic capacity	MS	3-way classification - reduction/no-change/increase with respect to previous assessment
T25-FW	A quantitative mobility and leg function performance test based on a timed 25 step walk	MS, Stroke	binary classification - classify between normal walking (T25-FW ≤ 4.4 s) and abnormal walking (T25-FW ≥ 4.4 s) - predict whether change ≥ 17.8% compared to previous assessment
Stabilometry	Use of a computerized platform for stabilometric (body sway), as well as posturometric (center of pressure during quiet standing) examination	MS	binary classification - normal (sway area ≤ 200 mm${}^{2}$) - abnormal (sway area > 200 mm${}^{2}$)
Romberg Test	Test to asses the patient’s balance using joint position, proprioception, and vestibular stimuli without visual aid	Stroke	binary classification - positive (existence of proprioception disorder) - negative (minimal or no swaying)
Berg Balance Scale	Determine patient’s ability to safely balance during a series of 14 predetermined tasks, each having a 0–4 ordinal rating	PD	binary classification - fall risk (BBS < 52) versus no fall risk (BBS ≥ 52) - change ≥ 3 points compared to previous assessment
		MS	binary classification - fall risk (BBS < 45) versus no fall risk (BBS ≥ 45) - change ≥ 3 points compared to previous assessment
		Stroke	binary classification - fall risk (BBS < 44) versus no fall risk (BBS ≥ 44) - change ≥ 3 points compared to previous assessment
NHPT	9-HPT is a brief, standardized, quantitative test of upper extremity function	MS	3-way classification - normal function (NHPT ≤ 18 s) - had dysfunction (18 s < NHPT ≤ 33.2 s) - severe dysfunction (NHPT > 33.2 s) binary classification - change ≥ 4.38 s compared to previous assessment
		Stroke	3-way classification - normal function (NHPT ≤ 18 s) - had dysfunction (18 s < NHPT ≤ 33.2 s) - severe dysfunction (NHPT > 33.2 s) binary classification - change ≥ 32.8 s compared to previous assessment
MRC	Quantify the muscle strength of a particular muscle group in relation to the movement of a single joint	MS, Stroke	6-way classification - 0: No movement—0/5 MRC - 1: Flicker of movement—1/5 MRC - 2: Through full range actively with gravity counterbalanced—2/5 MRC - 3: Through full range actively against gravity—3/5 MRC - 4: Through full range actively against some resistance—4/5 MRC - 5: Through full range actively against strong resistance—5/5 MRC
Modified Ashworth	Measure spasticity in patients who suffered a stroke	MS, Stroke	5-way classification of muscle tone - 0: no increase - 1: slight increase - 2: marked increase - 3: considerable increase - 4: affected parts rigid in flexion or tension
MDS-UPDRS II	Standard PD scale of report of activities of daily living (ADL) based on motor activity	PD	binary classification - increase of >6 points compared to a previous assessment
**Domain I—Mobility, general motor or physical function**			
MDS-UPDRS III	Standard PD scale of motor performance	PD	binary classification - increase of >6 points compared to a previous assessment
MDS-UPDRS IV	Standard PD scale of motor fluctuations and dyskinesias	PD	binary classification - increase of >2 points compared to a previous assessment
HOEHN YAHR	PD scale measuring global motor function by report	PD	binary classification - increase of >1 points compared to a previous assessment
SCOPA-Autonomic	PD scale measuring various aspects of autonomic function	PD	binary classification - increase of >10 points compared to a previous assessment
Laboratory Investigation of Autonomic Function	Various neurophysiological tests to assess the sympathetic and parasympathetic responses to various stimuli	PD	binary classification - increase of 20% compared to a previous assessment
Daily rating of motor complications	Relevant questions about motor complications (dyskinesias and off periods) reported by the patient	PD	binary classification - predict 2 point increase in devised scale
Diary of motor condition	Recording of severity level of dyskinesias and off periods, at 30 min intervals, during the intense monitoring period	PD	binary classification - predict 20% increase in off time or dyskinesias
EDSS	The Expanded Disability Status Scale (EDSS) quantifies disability in multiple sclerosis and monitors changes in disability level over time	MS	4-way classification - minimal disability (0–2.5) - mild disability (3–5.5) - moderate disability (6–7.5) - severe disability (>7.5) binary classification - change vs. no change compared to previous milestone

**Table A5:** Prediction targets and ground-truth conditions for medical tests in cognitive domain.

Variable	Description	Pilot Study	Prediction Target and Condition
**Domain III—Mental and cognitive ability**			
MDS-UPDRS I	Standard PD scale of non-motor symptoms, such as cognitive, emotional, autonomic, sleep, fatigue or pain issues	PD	binary classification - predict an increase of ≥6 points compared to previous measurement
SDMT	The symbol digit modalities test (SDMT) evaluates attention, processing speed and visual scanning	PD, MS	binary classification - normal (SDMT ≥ 41) vs. abnormal (SDMT < 41) - predict change of ≥17.1%
BVMT-R	Screening test for visual and spatial memory	PD, MS	binary classification - normal (score ≥ 22) vs. abnormal (score < 22)
CVLT-II	Measure of verbal learning and memory	PD, MS	binary classification - normal (score ≥ 47) vs. abnormal (score < 47)
Verbal Fluency	Short test of verbal functioning. Performance influenced by cognitive processes, mainly including attention-executive functioning, episodic memory, and language	PD, MS	binary classification - normal (score ≥ 21) vs. abnormal (score < 21) - predict change of ≥10 points
MOCA	Measures global cognitive function, with an emphasis on executive frontal lobe functions	MS, Stroke	3-way classification - normal (score ≥ 26) - abnormal (20 ≤ score < 26) - dementia (score < 20) binary classification - predict change of ≥4 points
FAST	Functional scale evaluating patients at the more moderate-severe stages of dementia when the MMSE no longer can reflect changes in a meaningful clinical way	Stroke	7-way classification 1. normal adult 2. normal older adult 3. early dementia 4. mild dementia 5. moderate dementia 6. moderately severe dementia 7. severe dementia
Virtual Supermarket Test	A serious game-based test to assess global cognition, executive function and visuo-spatial abilities (see Section 2.2.2)	PD, MS, Stroke	binary classification - predict significant worsening of performance as scored by test

**Table A6:** Prediction targets and ground-truth conditions for medical tests in sleep, emotional status and quality-of-life domains.

Variable	Description	Pilot Study	Prediction Target and Condition
**Domain II—Sleep disorders**			
Polysomnography	Video, EEG, EMG, EKG, breathing and heart rate monitoring to assess sleep	PD	binary classification - predict increase of ≥25% in any pathological pattern (insomnia, RBD, heart rate, etc.) - predict a ≥4% drop of oxygen saturation or a level below 88%
PD Sleep Scale	Questionnaire measuring various aspects of sleep and wakefulness related to somnolence	PD	binary classification - predict a drop of ≥25 points
Epworth Sleepiness Scale, Athens Insomnia Scale	Scales estimating daytime sleepiness and insomnia	PD	binary classification - predict an increase of ≥6 points compared to previous measurement
RBD Questionnaire	Measure the possibility of the existence of REM sleep behavior disorder	MS, PD	binary classification - predict increase of ≥4 points
**Domain IV—Emotional status**			
Geriatric Depression Scale	Questionnaire measuring the degree of depressive symptomatology	PD	binary classification - predict worsening by ≥3 points compared to previous measurement
Beck Anxiety Index	Questionnaire measuring anxiety	PD, MS	binary classification - predict worsening by ≥10 points compared to previous measurement
PHQ-9	Monitor the severity of depression and response to the treatment, supporting a diagnosis of depression for patients having suffered from a stroke	MS, Stroke	5-way classification - none (score 0–4) - mild (score 5–9) - moderate (score 10–14) - moderately sever (score 15–19) - severe (score 20–27)
**Domain V—Quality of life and daily living**			
PDQ39 or PDQ8	Gestalt quality-of-life measure, with 8 or 39 questions probing the impact of motor and non-motor aspects on patient quality of life	PD, MS	binary classification - predict worsening by >10 points compared to previous measurement
Schwabb & England	Scale measuring disease impact on activities of daily living	PD, MS	binary classification - predict decline of metric compared to previous measurement
Radboud Dysarthria Assessment (RDA)	Standard set of common speech and maximum performance (speech-like) tasks for the perceptual analysis of speech, using a qualitative recording form, a severity scale and a self-evaluation questionnaire	Stroke	6-way classification - no dysarthria - minimal dysarthria - mild dysarthria - mild/severe dysarthria - severe dysarthria - very severe dysarthria
NIHSS scale	Scale to objectively quantify the impairment caused by a stroke	Stroke	5-way classification - no stroke symptoms (0) - minor stroke (1–4) - moderate stroke (5–15) - moderate to severe stroke (16–20) - severe stroke (21–42)
MRS	Measure the degree of disability or dependence in the daily activities of people who have suffered a stroke	Stroke	6-way classification - no symptoms - no significant disability - slight disability - moderate disability - moderately severe disability - severe disability
Barthel Index	Ordinal scale used to measure performance in activities of daily living	MS, Stroke	4-way classification - total dependency (0–20) - severe dependency (21–60) - moderate dependency (61–90) - slight dependency (91–99)
